# Hepatobiliary adverse-event reporting patterns for endocrine therapies in breast cancer: a pharmacovigilance study based on FAERS and VigiAccess

**DOI:** 10.3389/fendo.2026.1940744

**Published:** 2026-10-23

**Authors:** Weiguo Mao, Yonghui Liu, Ben Liu, Bin Zhu

**Affiliations:** 1 Department of General Surgery, Changshu No. 2 People’s Hospital, Affiliated Hospital of Nantong University, Suzhou, Jiangsu, China; 2 Department of Laboratory Medicine, Nanjing Zhongda Hospital, School of Medicine, Southeast University, Nanjing, Jiangsu, China; 3 Pediatric Intensive Care Unit, Yancheng First Hospital, Affiliated Hospital of Nanjing University Medical School, Yancheng, Jiangsu, China; 4 Department of General Surgery, Yancheng First Hospital, Affiliated Hospital of Nanjing University Medical School, Yancheng, Jiangsu, China

**Keywords:** breast cancer, disproportionality analysis, endocrine therapy, FAERS, hepatobiliary adverse events, pharmacovigilance, VigiAccess

## Abstract

**Background:**

Endocrine therapy is central to the treatment of hormone receptor-positive breast cancer, yet hepatobiliary adverse-event reporting across endocrine agents remains incompletely characterized. We evaluated hepatobiliary reporting patterns and disproportionality signals using FAERS, with VigiAccess as a supplementary data source.

**Methods:**

FAERS data from Q1–2004 to Q1–2026 were analyzed for seven endocrine therapies: tamoxifen, toremifene, anastrozole, letrozole, exemestane, fulvestrant, and elacestrant. Hepatobiliary adverse events were identified using prespecified MedDRA Preferred Terms (PTs). Drug-level disproportionality was assessed using reporting odds ratios (RORs) and information components (ICs) within a FAERS reference population restricted by breast cancer indication and primary-suspect drug role. PT- and HLT-level reporting patterns, class-level differences, sensitivity analyses, and time-to-onset (TTO) characteristics were also explored. VigiAccess was used for a non-equivalent exploratory disproportionality comparison based on aggregated adverse-event term counts.

**Results:**

The FAERS hepatobiliary cohort comprised 2,630 unique case reports meeting the prespecified target-drug, breast cancer indication, primary-suspect, and hepatobiliary adverse-event criteria. Toremifene showed the largest disproportionality estimate [ROR = 5.91, 95% CI: 4.55–7.68; IC = 2.45, IC025 = 1.96], with positive signals also observed for letrozole, exemestane, and fulvestrant. Tamoxifen, anastrozole, and elacestrant did not meet the predefined positive-signal criteria. Signals for toremifene, letrozole, and exemestane were robust across sensitivity analyses, whereas the weak fulvestrant signal was more sensitive to alternative endpoint definitions, drug-role classifications, and clinical-context exclusions. The VigiAccess analysis showed concordance for the prominent toremifene signal but discordant findings for tamoxifen, letrozole, and exemestane. PT- and HLT-level analyses showed exploratory differences in reported hepatobiliary event distributions across drugs and therapy classes. TTO analyses suggested an earlier concentration of reported onset times at the class level, although within-class heterogeneity and incomplete timing data limited interpretation.

**Conclusions:**

This pharmacovigilance study identified heterogeneous hepatobiliary adverse-event reporting patterns and disproportionality signals across endocrine therapies used in breast cancer. Toremifene, letrozole, and exemestane showed consistent signals across multiple sensitivity analyses, whereas other agents demonstrated more context-dependent patterns. These findings should be regarded as hypothesis-generating pharmacovigilance evidence rather than estimates of clinical incidence, comparative safety, or causal hepatotoxicity and require confirmation in clinical and pharmacoepidemiologic studies.

## 1 Introduction

Breast cancer remains a major global health burden among women. According to the latest GLOBOCAN estimates, female breast cancer ranks among the most commonly diagnosed malignancies worldwide and accounts for a substantial proportion of global cancer incidence (1). Hormone receptor-positive disease accounts for the majority of breast cancers, and endocrine therapy remains a cornerstone of systemic treatment for estrogen receptor-positive disease (2, 3). Major endocrine therapy strategies include selective estrogen receptor modulators (SERMs), aromatase inhibitors (AIs), and selective estrogen receptor degraders (SERDs), which modulate estrogen signaling through receptor modulation, suppression of estrogen synthesis, or receptor degradation, respectively (4). Large patient-level meta-analyses have demonstrated the clinical benefit of endocrine therapy, including reduced recurrence with AIs compared with tamoxifen in early breast cancer and substantial reductions in long-term recurrence and breast cancer mortality with approximately five years of adjuvant tamoxifen (5, 6). Given the prolonged duration of endocrine therapy, long-term treatment-related adverse effects warrant attention alongside antitumor efficacy.

Although endocrine therapies are generally well tolerated, adverse-event reporting patterns vary across drug classes and individual agents. Previous studies have characterized a broad range of treatment-related adverse effects across breast cancer therapies, including metabolic, musculoskeletal, cardiovascular, and other complications (7). Recent real-world studies have further highlighted heterogeneity in long-term treatment-related outcomes across breast cancer subgroups and clinical profiles (8). Hepatobiliary events warrant particular attention because of the liver’s central role in drug metabolism, hormone regulation, lipid metabolism, and bile acid homeostasis. Reported hepatobiliary events during endocrine therapy may include liver enzyme abnormalities, hepatic steatosis, cholestasis, drug-induced liver injury, hepatic failure, and vascular complications. Clinical and observational studies have reported fatty liver disease, steatohepatitis, and drug-induced liver injury during tamoxifen treatment, while liver-function abnormalities have also been described with aromatase inhibitors and toremifene (9, 10). Post-marketing pharmacovigilance studies have characterized adverse-event reporting patterns for individual endocrine agents, including third-generation AIs, elacestrant, and other SERDs (11, 12). Recent breast cancer pharmacovigilance studies have also used complementary spontaneous-reporting databases to characterize adverse-event reporting signals across anticancer therapies, illustrating both the value and the interpretive limitations of real-world pharmacovigilance data (13). Nevertheless, systematic comparisons of hepatobiliary adverse-event reporting patterns across commonly used endocrine therapies in breast cancer remain limited.

Spontaneous-reporting systems are widely used to detect rare, delayed, or unexpected post-marketing safety signals. The FDA Adverse Event Reporting System (FAERS) contains individual case safety reports with information on suspected drugs, adverse events, indications, reporter characteristics, outcomes, and time-related variables and is widely used for pharmacovigilance signal detection (13). VigiAccess, a public interface to the WHO global pharmacovigilance database, provides aggregated adverse-event reporting information and can support supplementary exploratory comparisons. However, spontaneous-reporting systems are subject to underreporting, reporting bias, incomplete data, potential duplicate reporting, and the absence of reliable exposure denominators; consequently, disproportionality findings should be interpreted as reporting signals rather than as estimates of incidence or causal effects (14–16). In this study, we systematically evaluated hepatobiliary adverse-event reporting across seven endocrine therapies used in breast cancer. Using FAERS as the primary data source, we assessed drug-level disproportionality signals, PT- and HLT-level reporting patterns, class-level differences, factors associated with hepatobiliary adverse-event reporting, sensitivity analyses, and time-to-onset characteristics. VigiAccess was used as a supplementary source for a non-equivalent exploratory comparison. The study was designed to characterize pharmacovigilance signals and reported-event distributions rather than to estimate incidence, comparative clinical safety, or causal hepatotoxicity.

## 2 Materials and methods

### 2.1 Study design and data sources

This retrospective pharmacovigilance study used the FDA Adverse Event Reporting System (FAERS) as the primary data source. FAERS is a publicly available spontaneous reporting database for post-marketing pharmacovigilance that contains individual case safety reports submitted by healthcare professionals, consumers, manufacturers, and other sources. Quarterly FAERS data from Q1–2004 through Q1–2026 were analyzed. The DEMO, DRUG, REAC, OUTC, RPSR, THER, and INDI files were used to extract demographic characteristics, reported drug information, adverse events, outcomes, report sources, therapy dates, and indications. Duplicate reports were removed using the FDA-recommended procedure: for records sharing the same CASEID, the report with the most recent FDA_DT was retained, and when FDA_DT was identical, the record with the highest PRIMARYID was kept. Reports listed in the deleted case files were excluded. Because FAERS data are publicly available and anonymized, ethics approval and informed consent were not required. This study was reported with reference to the READUS-PV recommendations for disproportionality analyses based on individual case safety reports (17).

VigiAccess was used as a supplementary pharmacovigilance data source. Data were retrieved from the public VigiAccess interface on July 7, 2026 using a Python-based extraction procedure. Aggregated adverse-event term counts were obtained separately for tamoxifen, toremifene, anastrozole, letrozole, exemestane, fulvestrant, and elacestrant and organized according to MedDRA Preferred Terms (PTs) and System Organ Classes (SOCs). Hepatobiliary adverse events were identified using the MedDRA SOC “Hepatobiliary disorders.” For each target endocrine therapy, a four-cell contingency table was constructed from the available aggregated counts: *a* represented hepatobiliary adverse-event counts for the target therapy, *b* represented non-hepatobiliary adverse-event counts for the target therapy, *c* represented hepatobiliary adverse-event counts for the remaining six endocrine therapies combined, and *d* represented non-hepatobiliary adverse-event counts for the remaining six endocrine therapies combined. Thus, the comparator background comprised the other endocrine therapies included in this study rather than all medicinal products in VigiAccess. ROR was calculated as *ad/bc*, with 95% confidence intervals derived using the logarithmic approximation. IC was calculated as log2[*a(a+b+c+d)/((a+b)(a+c))*], using the same observed-to-expected framework as in the FAERS analysis. The exact four-cell counts and derived disproportionality estimates are provided in **Supplementary Table 12**. Because VigiAccess provides aggregated term-level counts rather than individual case-level reports, indication-restricted, drug-role-restricted, adjusted, concomitant-medication, subgroup, and time-to-onset analyses were not feasible. Accordingly, the VigiAccess analysis was treated as a non-equivalent supplementary exploratory comparison rather than as a direct replication or external validation of the FAERS analysis.

### 2.2 Drug identification and exposure definition

Seven endocrine therapies commonly used in breast cancer were included: tamoxifen, toremifene, anastrozole, letrozole, exemestane, fulvestrant, and elacestrant. Drugs were identified in the DRUG file using generic names, brand names, salt forms, and common synonyms and were grouped by active ingredient. The primary FAERS analyses were based on reports in which the target endocrine therapy was recorded as the primary suspect (PS) drug. To assess robustness to drug-role classification, disproportionality analyses were additionally repeated using PS+SS reports and all reported drug roles (PS, SS, concomitant, and interacting). All other analytic definitions, including the study period, breast cancer indication restriction, hepatobiliary endpoint, comparator framework, and signal criteria, were unchanged across the three drug-role analyses. Breast cancer-related reports were identified from the INDI file using prespecified indication terms, including “breast cancer,” “breast carcinoma,” “breast neoplasm malignant,” “metastatic breast cancer,” “breast cancer metastatic,” and “malignant neoplasm of breast” (**Supplementary Table 1**). Unique FAERS case reports meeting the target-drug, breast cancer indication, PS drug-role, and hepatobiliary adverse-event criteria formed the descriptive hepatobiliary cohort. For disproportionality analyses, the reference population additionally included non-hepatobiliary reports meeting the same breast cancer indication, PS drug-role, and study-period restrictions, with other PS drugs serving as the comparator background.

### 2.3 Definition of hepatobiliary adverse events

Hepatobiliary adverse events were identified from Preferred Terms (PTs) in the FAERS REAC file mapped to the MedDRA version 28.1 System Organ Class (SOC) “Hepatobiliary disorders.” All 227 PTs within this SOC were included in the broad hepatobiliary endpoint and further mapped to their corresponding high-level terms (HLTs) for hierarchical signal profiling. The broad endpoint was defined solely by membership in this MedDRA SOC, without manual selection of individual PTs. The complete PT list, including PT codes and HLT mappings, is provided in machine-readable form in **Supplementary Table 2**. To assess robustness under a more specific endpoint definition, a hepatic-injury sensitivity endpoint was constructed by intersecting the broad 227-PT set with ACTIVE, NARROW-scope PTs from the Drug related hepatic disorders Standardised MedDRA Query (SMQ) and retaining terms representing drug-induced liver injury, hepatocellular injury/hepatitis, cholestatic injury/hepatic-origin jaundice, or mixed liver injury. Nonspecific laboratory abnormalities, structural or mass/lesion terms, neoplasm- or progression-related manifestations, vascular/coagulation complications, steatotic or metabolic liver disorders, conditions with specific alternative etiologies, and terms denoting suspected rather than directly specified injury were excluded. The final restricted endpoint comprised 16 PTs, listed with their clinical categories and inclusion rationale in **Supplementary Table 3**.

### 2.4 Descriptive and disproportionality analyses

Descriptive analyses summarized age, sex, reporting year, reporter country, reporter occupation, seriousness, recorded clinical outcomes, target endocrine therapy, therapy class, and concomitant ovarian function suppression (OFS) use. Age was categorized *a priori* as ≤45, 45–60, and >60 years to provide clinically interpretable strata while preserving adequate report counts; missing age was classified as unknown descriptively and excluded from complete-case regression analyses. Seriousness and clinical outcomes were identified from the OUTC file. Clinical outcomes recorded in FAERS were treated as reported case outcomes and were not considered causally attributable to the endocrine therapy or hepatobiliary event. In particular, a recorded death indicated only that death was reported in the case. Death-related outcomes recorded in FAERS were descriptively assessed. Additional indicators, including malignancy progression/recurrence and hepatic metastasis/neoplasm among death-reported cases, were summarized separately. A sensitivity analysis excluding target-drug reports with death recorded as an outcome was prespecified to evaluate the influence of death-related reports on drug-level disproportionality estimates. Concomitant OFS therapy was identified from gonadotropin-releasing hormone agonists recorded in the DRUG file, including leuprolide, goserelin, and triptorelin.

Drug-level disproportionality analyses were conducted within a restricted FAERS reference population comprising breast cancer reports from Q1–2004 to Q1–2026 in which a drug was recorded as the primary suspect (PS) (18, 19). No sex restriction was applied. For each target endocrine therapy, a 2 × 2 contingency table was constructed at the adverse-event-record level. Specifically, *a* represented hepatobiliary adverse-event records associated with the target drug recorded as the primary suspect (PS); *b* represented non-hepatobiliary adverse-event records associated with the target drug recorded as PS; *c* represented hepatobiliary adverse-event records associated with other PS drugs within the same restricted reference population; and *d* represented non-hepatobiliary adverse-event records associated with other PS drugs within the same restricted reference population. Thus, comparator records were subject to the same study-period, breast cancer indication, and PS drug-role restrictions as target-drug records. Disproportionality was assessed using the reporting odds ratio (ROR) and information component (IC). The corresponding formulas and predefined signal criteria are provided in **Supplementary Table 4**. A positive signal required *a* ≥3, a lower 95% confidence limit for the ROR >1, and IC025 >0, where IC025 denotes the lower bound of the 95% credible interval for the IC (20). For visualization of PT-level signal strength, drug–event pairs that satisfied all predefined positive-signal criteria (*a* ≥3, lower 95% confidence limit of the ROR >1, and IC025 >0) were further categorized according to ROR magnitude as weak (ROR <5), strong (ROR 5–30), or very strong (ROR >30), following a previously published pharmacovigilance classification approach (21). Drug–event pairs not satisfying all positive-signal criteria were classified as no signal.

Drug-, PT-, and HLT-level disproportionality analyses were performed to characterize hepatobiliary reporting patterns across individual drugs and therapy classes. Drug-level analyses were repeated using the restricted 16-PT hepatic-injury endpoint, with all other analytic definitions unchanged from the primary broad-endpoint analysis. Robustness to drug-role classification was assessed by independently rerunning the drug-level disproportionality analyses under PS-only, PS+SS, and all-role definitions, while retaining the same study period, breast cancer indication restriction, hepatobiliary adverse-event definition, comparator framework, and prespecified signal criteria.

Potential advanced-disease burden was assessed using metastatic or stage IV breast cancer indication markers recorded in the INDI file, while liver-specific disease burden was assessed using liver metastasis/hepatic neoplasm indicators recorded in the INDI file. Because explicit disease-stage information was sparsely recorded and treatment line could not be reliably reconstructed from FAERS, these variables were used as descriptive disease-burden indicators rather than formal adjustment covariates. Concomitant anticancer therapies were identified from the DRUG file using PRIMARYID. CDK4/6 inhibitor use was defined by recorded palbociclib, ribociclib, abemaciclib, or dalpiciclib, while concomitant cytotoxic chemotherapy and HER2-targeted therapy were identified from other recorded anticancer medications. Their prevalence was summarized among hepatobiliary reports for each endocrine therapy. Separate exclusion sensitivity analyses were then performed after removing target-drug reports containing liver metastasis/hepatic neoplasm indicators or concomitant CDK4/6 inhibitors, cytotoxic chemotherapy, or HER2-targeted therapy. For each exclusion scenario, target-drug counts were recalculated while retaining the primary-analysis comparator background, and ROR and IC estimates were recomputed using the same prespecified signal-detection framework.

### 2.5 Comparative analysis and factors associated with hepatobiliary adverse-event reporting

To explore class-level differences in hepatobiliary Preferred Term (PT) reporting, pairwise comparisons were performed among aromatase inhibitors (AIs), selective estrogen receptor modulators (SERMs), and selective estrogen receptor degraders (SERDs). For each pairwise comparison, the analysis population comprised eligible breast cancer reports involving either of the two endocrine therapy classes being compared under the primary-suspect (PS) drug-role definition. Reports involving more than one target endocrine therapy were excluded from the pairwise class comparisons to avoid assigning the same report to both comparison groups. For each eligible PT, the dependent variable was whether that PT was reported in an individual report (1 = present; 0 = absent), and endocrine therapy class was the primary explanatory variable. Multivariable logistic regression models were fitted using complete-case data and adjusted for age group, sex, reporting year, and concomitant ovarian function suppression (OFS) use. Only PTs with at least 10 events overall and at least 3 events in each comparison group were analyzed. Adjusted odds ratios (ORs) with 95% confidence intervals (CIs) were estimated. Raw P values were adjusted using the Benjamini–Hochberg false-discovery-rate procedure separately within each pairwise comparison, with q < 0.05 considered statistically significant.

To explore factors associated with overall hepatobiliary adverse-event reporting, separate univariable and multivariable logistic regression analyses were performed within the AI, SERD, and SERM classes. The dependent variable was the presence of at least one prespecified hepatobiliary PT in an individual report (1 = present; 0 = absent); therefore, both hepatobiliary and non-hepatobiliary reports within the same endocrine therapy class were included in the analysis population. Age group, sex, and concomitant OFS use were entered simultaneously into each multivariable model. Age was categorized *a priori* as ≤45, 45–60, and >60 years, with 45–60 years as the reference category; female sex and no concomitant OFS use were used as the reference categories for sex and OFS status, respectively. Complete-case analysis was applied, and reports with missing values for variables required in the respective models were excluded. The final numbers of hepatobiliary event/non-event reports included in the analyses were 1,449/19,471 for AIs, 367/4,973 for SERDs, and 167/2,678 for SERMs.

### 2.6 Time-to-onset analysis

Time-to-onset (TTO) analysis was performed among unique hepatobiliary case reports with evaluable treatment-start and adverse-event onset dates. Treatment-start dates were obtained from the THER file and adverse-event onset dates from the DEMO file. TTO was defined as the number of days between the recorded treatment-start date and adverse-event onset date. Reports with missing or incomplete dates or with adverse-event onset preceding treatment initiation were excluded. Same-day onset (TTO = 0 days) was retained in descriptive summaries and treated as 1 day for Weibull fitting. No arbitrary upper TTO cutoff was imposed because endocrine therapy may be administered for prolonged periods and spontaneous-report data cannot reliably distinguish true long-latency events from delayed documentation; long intervals were therefore retained but interpreted cautiously. The derivation of the TTO analysis cohort, including exclusion criteria for missing or invalid timing information, is illustrated in **Supplementary Figure 1**. Valid-TTO completeness was assessed by individual drug and therapy class. Potential selection bias related to timing-data availability was evaluated by comparing reports included in the TTO analysis with excluded reports according to prespecified clinical and reporting characteristics. For reports with multiple target-drug treatment-start records, a sensitivity analysis using the most recent complete start date preceding adverse-event onset was performed and did not materially alter the class-level TTO estimates. Differences in TTO distributions among therapy classes were assessed using non-parametric tests. Weibull distribution analysis was used to characterize temporal reporting patterns; an upper 95% confidence limit for the shape parameter β < 1 indicated an early-failure pattern, a 95% confidence interval including 1 indicated a random-failure pattern, and a lower 95% confidence limit >1 indicated a wear-out pattern (22). Because TTO information was available for only a subset of reports, these analyses were interpreted as descriptive assessments of reported onset timing rather than estimates of a biological risk period.

### 2.7 Statistical analysis

All statistical analyses were performed using R software. Categorical variables were summarized as frequencies and percentages, and continuous variables as medians and interquartile ranges. Disproportionality analyses were based on the reporting odds ratio (ROR) and information component (IC) using the prespecified signal criteria. Time-to-onset distributions were compared using non-parametric tests, and univariable and multivariable logistic regression models were used to estimate crude and adjusted odds ratios with 95% confidence intervals. For inferential analyses not subject to multiple-testing correction, a two-sided P value < 0.05 was considered statistically significant. For PT-level pairwise comparisons among endocrine therapy classes, raw P values were adjusted separately within each pairwise comparison using the Benjamini–Hochberg false-discovery-rate procedure, with q <0.05 considered statistically significant. PT- and HLT-level disproportionality analyses were treated as exploratory pharmacovigilance signal-screening analyses and evaluated using the prespecified ROR and IC criteria rather than conventional P-value-based hypothesis testing. Given the spontaneous-reporting nature of FAERS, findings were interpreted as reporting patterns or pharmacovigilance signals rather than estimates of incidence, comparative safety, or causal effects.

## 3 Results

### 3.1 Population characteristics and descriptive analysis

From Q1–2004 to Q1 2026, 24,389,966 safety reports were retrieved from FAERS. After removal of 4,063,184 duplicate reports, 20,326,782 unique reports remained. After applying the predefined drug, indication, primary-suspect, and hepatobiliary adverse-event criteria, 2,630 unique FAERS case reports were included in the descriptive hepatobiliary cohort (**Figure 1**). These unique case reports involved tamoxifen (n = 154), toremifene (n = 51), anastrozole (n = 451), letrozole (n = 1,139), exemestane (n = 296), fulvestrant (n = 516), and elacestrant (n = 23) as primary-suspect drugs. Reports involving patients aged >60 years constituted the largest age group (n = 995, 37.8%), followed by those aged 45–60 years (n = 674, 25.6%); age was unknown in 639 reports (24.3%). Most reports involved female patients (n = 2,505, 95.2%) (**Table 1**). Hepatobiliary report counts were relatively stable from 2004 to 2017 and increased after 2018, reaching their highest annual count in 2025; the lower count in 2026 reflects availability of only first-quarter data (**Figure 2**). Reports from countries other than the four individually summarized countries accounted for 65.6% (n = 1,725); among individually reported countries, Germany accounted for 13.4% (n = 352), the United States 11.6% (n = 306), Canada 4.9% (n = 128), and the United Kingdom 4.5% (n = 119). Concomitant OFS use was recorded in 5.4% of reports (**Table 1**).

**Figure 1:**
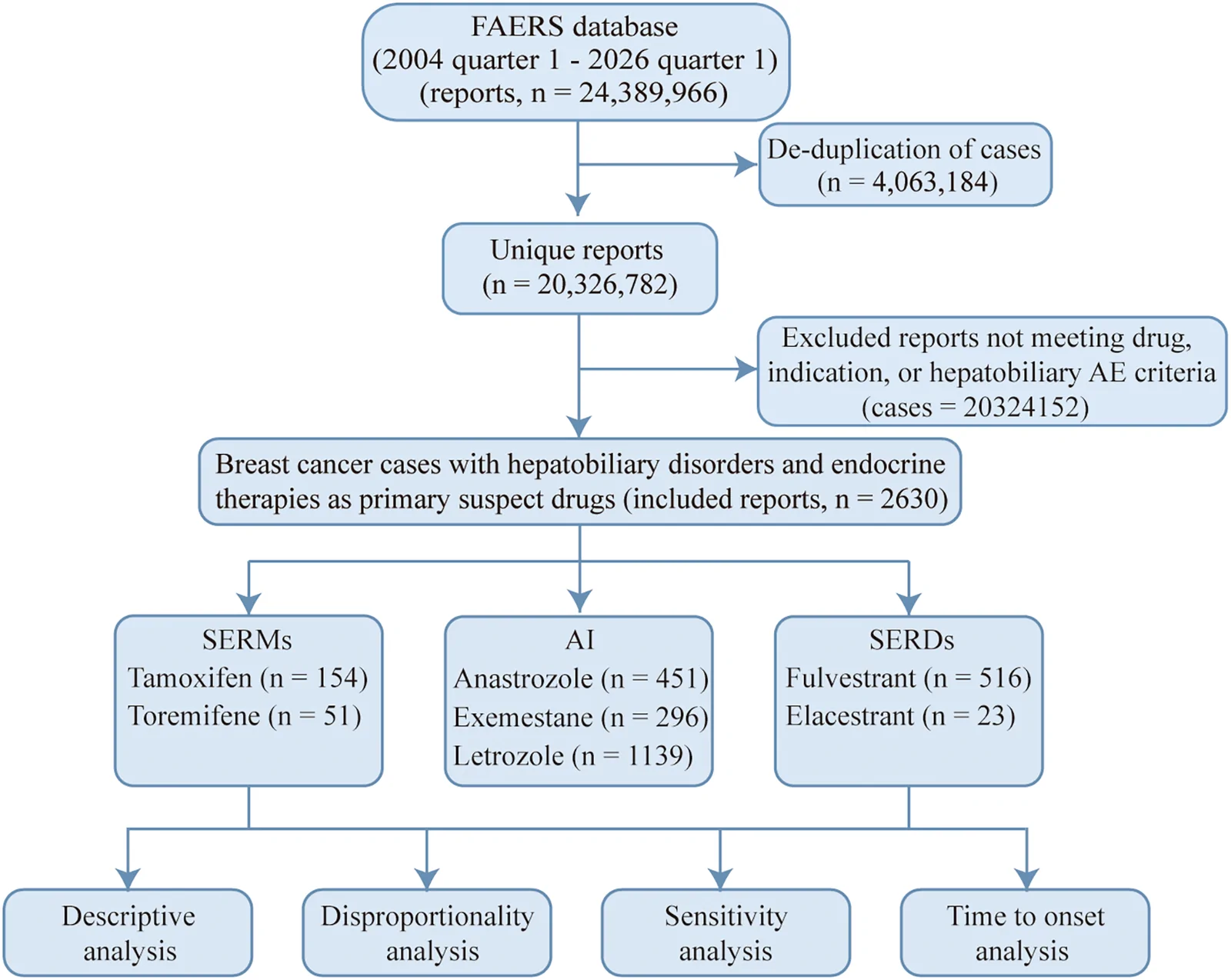
Flowchart of FAERS case report selection. FAERS case reports from Q1–2004 to Q1–2026 were screened according to target drug, breast cancer indication, primary-suspect drug role, and hepatobiliary adverse-event criteria. A total of 2,630 unique FAERS case reports were included in the descriptive hepatobiliary cohort.

**Table 1:** Characteristics of unique FAERS hepatobiliary case reports associated with endocrine therapies.

Characteristics	Overall N = 2630	Tamoxifen N = 154	Toremifene N = 51	Letrozole N = 1139	Anastrozole N = 451	Exemestane N = 296	Fulvestrant N = 516	Elacestrant N = 23
Age groups								
≤45	322 (12.2%)	38 (24.7%)	1 (2.0%)	128 (11.2%)	64 (14.2%)	13 (4.4%)	77 (14.9%)	1 (4.3%)
45-60	674 (25.6%)	57 (37.0%)	4 (7.8%)	293 (25.7%)	89 (19.7%)	97 (32.8%)	134 (26.0%)	0 (0%)
> 60	995 (37.8%)	46 (29.9%)	22 (43.1%)	452 (39.7%)	194 (43.0%)	125 (42.2%)	156 (30.2%)	0 (0%)
Unknown	639 (24.3%)	13 (8.4%)	24 (47.1%)	266 (23.4%)	104 (23.1%)	61 (20.6%)	149 (28.9%)	22 (95.7%)
Sex								
Female	2505 (95.2%)	144 (93.5%)	34 (66.7%)	1108 (97.3%)	433 (96.0%)	283 (95.6%)	501 (97.1%)	2 (8.7%)
Male	13 (0.5%)	4 (2.6%)	0 (0%)	3 (0.3%)	1 (0.2%)	3 (1.0%)	2 (0.4%)	0 (0%)
Unknown	112 (4.3%)	6 (3.9%)	17 (33.3%)	28 (2.5%)	17 (3.8%)	10 (3.4%)	13 (2.5%)	21 (91.3%)
Reporting year								
2004-2006	105 (4.0%)	22 (14.2%)	7 (13.7%)	16 (1.4%)	27 (6.0%)	28 (9.5%)	5 (1.0%)	0 (0.0%)
2007-2009	142 (5.4%)	23 (14.8%)	8 (15.7%)	21 (1.8%)	42 (9.3%)	44 (14.9%)	4 (0.8%)	0 (0.0%)
2010-2012	148 (5.6%)	14 (9.0%)	7 (13.7%)	42 (3.7%)	46 (10.2%)	26 (8.8%)	13 (2.5%)	0 (0.0%)
2013-2015	153 (5.8%)	13 (8.4%)	6 (11.8%)	42 (3.7%)	40 (8.9%)	32 (10.8%)	20 (3.9%)	0 (0.0%)
2016-2018	227 (8.6%)	13 (8.3%)	8 (15.7%)	93 (8.2%)	51 (11.3%)	22 (7.4%)	40 (7.8%)	0 (0.0%)
2019-2021	530 (20.2%)	27 (17.5%)	4 (7.8%)	251 (22.0%)	79 (17.5%)	56 (18.9%)	113 (21.9%)	0 (0.0%)
2022-2024	814 (31.0%)	27 (17.5%)	6 (11.8%)	462 (40.6%)	72 (16.0%)	66 (22.3%)	179 (34.7%)	2 (8.7%)
2025-2026	511 (19.4%)	15 (9.7%)	5 (9.8%)	212 (18.6%)	94 (20.8%)	22 (7.4%)	142 (27.5%)	21 (91.3%)
Weight groups								
<50 kg	73 (2.8%)	4 (2.6%)	1 (2.0%)	35 (3.1%)	13 (2.9%)	11 (3.7%)	9 (1.7%)	0 (0%)
>100 kg	67 (2.5%)	8 (5.2%)	0 (0%)	40 (3.5%)	6 (1.3%)	3 (1.0%)	9 (1.7%)	1 (4.3%)
50-100kg	888 (33.8%)	49 (31.8%)	0 (0%)	401 (35.2%)	144 (31.9%)	116 (39.2%)	177 (34.3%)	1 (4.3%)
Unknown	1602 (60.9%)	93 (60.4%)	50 (98.0%)	663 (58.2%)	288 (63.9%)	166 (56.1%)	321 (62.2%)	21 (91.3%)
Reporter groups								
Consumer	407 (15.5%)	24 (15.6%)	11 (21.6%)	153 (13.4%)	69 (15.3%)	52 (17.6%)	78 (15.1%)	20 (87.0%)
Health professional	331 (12.6%)	11 (7.1%)	14 (27.5%)	157 (13.8%)	54 (12.0%)	31 (10.5%)	63 (12.2%)	1 (4.3%)
Pharmacist	221 (8.4%)	15 (9.7%)	1 (2.0%)	123 (10.8%)	38 (8.4%)	30 (10.1%)	13 (2.5%)	1 (4.3%)
Physician	1325 (50.4%)	65 (42.2%)	2 (3.9%)	614 (53.9%)	206 (45.7%)	127 (42.9%)	310 (60.1%)	1 (4.3%)
Missing	346 (13.2%)	39 (25.3%)	23 (45.1%)	92 (8.1%)	84 (18.6%)	56 (18.9%)	52 (10.1%)	0 (0%)
Reporter country								
United States	306 (11.6%)	20 (13.0%)	0 (0.0%)	65 (5.7%)	100 (22.2%)	49 (16.6%)	52 (10.1%)	20 (87.0%)
United Kingdom	119 (4.5%)	25 (16.2%)	0 (0.0%)	53 (4.7%)	14 (3.1%)	9 (3.0%)	18 (3.5%)	0 (0.0%)
Canada	128 (4.9%)	4 (2.6%)	0 (0.0%)	49 (4.3%)	29 (6.4%)	5 (1.7%)	41 (7.9%)	0 (0.0%)
Germany	352 (13.4%)	7 (4.5%)	0 (0.0%)	199 (17.5%)	41 (9.1%)	24 (8.1%)	81 (15.7%)	0 (0.0%)
Others	1725 (65.6%)	98 (63.6%)	51 (100.0%)	773 (67.9%)	267 (59.2%)	209 (70.6%)	324 (62.8%)	3 (13.0%)
Serious report								
Serious	2,222 (84.5%)	95 (61.7%)	28 (54.9%)	1059 (93.0%)	330 (73.2%)	199 (67.2%)	489 (94.8%)	22 (95.7%)
Non-serious	408 (15.5%)	59 (38.3%)	23 (45.1%)	80 (7.0%)	121 (26.8%)	97 (32.8%)	27 (5.2%)	1 (4.3%)
Outcomes groups								
Death	381 (14.5%)	7 (4.5%)	0 (0%)	135 (11.9%)	42 (9.3%)	32 (10.8%)	160 (31.0%)	5 (21.7%)
Disability	10 (0.4%)	2 (1.3%)	0 (0%)	2 (0.2%)	1 (0.2%)	4 (1.4%)	1 (0.2%)	0 (0%)
Hospitalization	601 (22.9%)	29 (18.8%)	10 (19.6%)	326 (28.6%)	91 (20.2%)	54 (18.2%)	79 (15.3%)	12 (52.2%)
Life-Threatening	111 (4.2%)	5 (3.2%)	0 (0%)	68 (6.0%)	14 (3.1%)	0 (0%)	24 (4.7%)	0 (0%)
Other	1522 (57.9%)	111 (72.1%)	36 (70.6%)	608 (53.4%)	303 (67.2%)	206 (69.6%)	252 (48.8%)	6 (26.1%)
Concurrent OFS use (yes)	141 (5.4%)	8 (5.2%)	0 (0%)	86 (7.6%)	13 (2.9%)	8 (2.7%)	26 (5.0%)	0 (0.0%)

Data are presented as n (%). Unique case reports were stratified by individual endocrine therapy, including tamoxifen, toremifene, letrozole, anastrozole, exemestane, fulvestrant, and elacestrant. OFS, ovarian function suppression.

**Figure 2:**
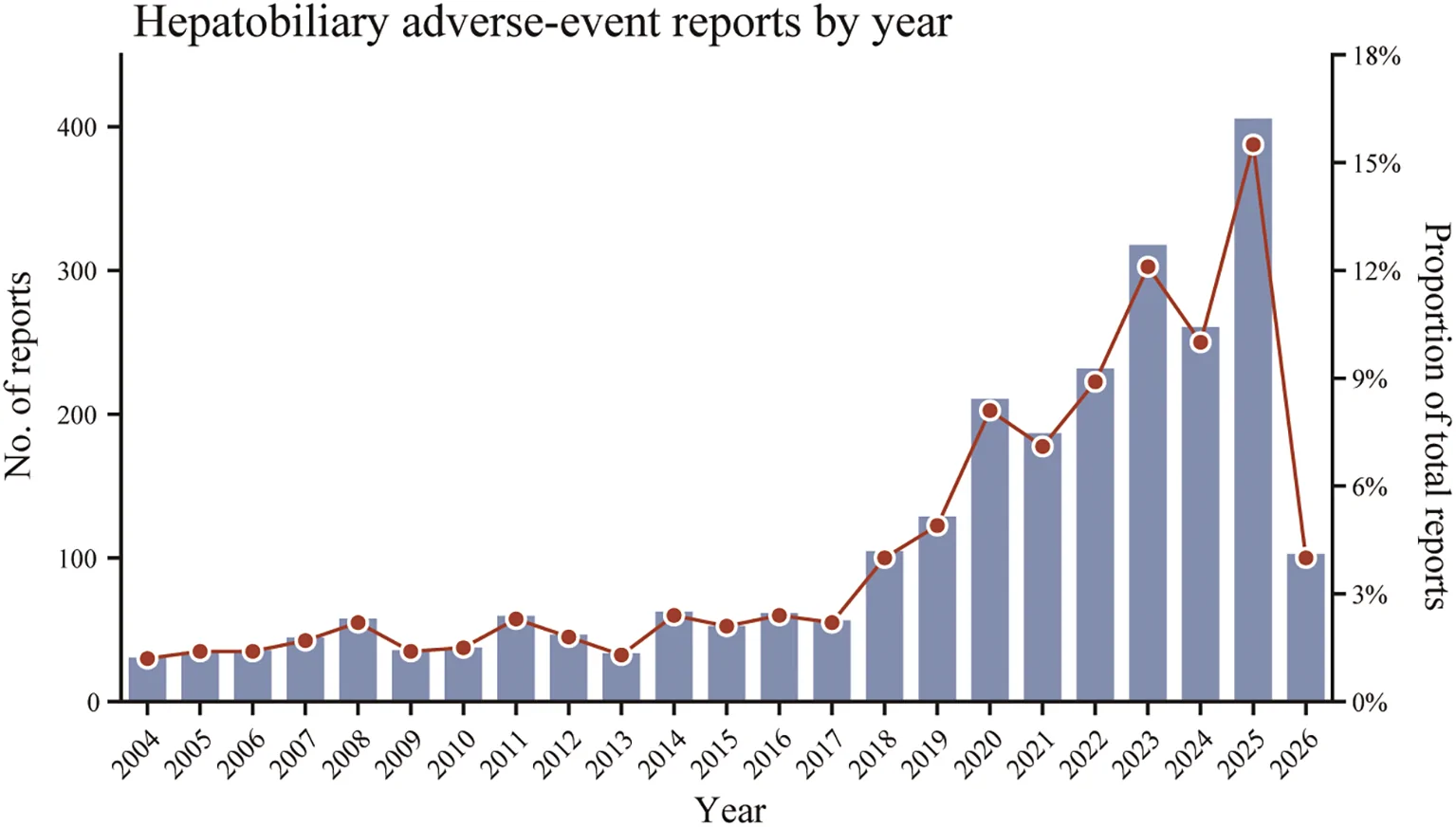
Annual trend of hepatobiliary adverse-event case reports. Bars indicate the annual number of hepatobiliary case reports, and the line indicates their proportion among all FAERS case reports in the corresponding year. Data for 2026 include only the first quarter.

Among the 2,630 hepatobiliary adverse-event reports included in the descriptive cohort, 2,222 (84.5%) were classified as serious reports. Death was recorded as an outcome in 381 reports (14.5%). The highest proportions of death-recorded cases were observed for fulvestrant [160/516 (31.0%)] and elacestrant [5/23 (21.7%)], although the latter estimate was based on a limited number of reports. Among death-recorded cases, 150 (39.4%) contained indicators of malignancy progression or recurrence and 100 (26.2%) contained hepatic metastasis/neoplasm-related indicators; these categories were not mutually exclusive (**Supplementary Table 5**). These findings represent outcomes recorded in FAERS reports and do not establish causality between endocrine therapy, hepatobiliary events, and death.

### 3.2 Disproportionality analysis

Drug-level disproportionality signals within the prespecified FAERS reference population are summarized in **Table 2**, and the corresponding four-cell contingency table counts (a, b, c, and d) are provided in **Supplementary Table 6**. Among the seven endocrine therapies, letrozole contributed the largest number of hepatobiliary adverse-event records (n = 1,549), followed by fulvestrant (n = 712), anastrozole (n = 635), exemestane (n = 385), tamoxifen (n = 215), toremifene (n = 62), and elacestrant (n = 25). Toremifene showed the largest disproportionality estimate [ROR = 5.91, 95% CI: 4.55–7.68; IC = 2.45, IC025 = 1.96]. Positive signals were also observed for letrozole [ROR = 1.46, 95% CI: 1.39–1.54; IC = 0.50, IC025 = 0.42], exemestane [ROR = 1.29, 95% CI: 1.17–1.43; IC = 0.36, IC025 = 0.21], and fulvestrant [ROR = 1.14, 95% CI: 1.05–1.23; IC = 0.18, IC025 = 0.06]. Tamoxifen, anastrozole, and elacestrant did not meet the predefined positive-signal criteria.

**Table 2:** Drug-level disproportionality analysis of hepatobiliary adverse-event reporting in FAERS.

Drug	Class	Target-drug adverse-event records, n (a+b)	Hepatobiliary adverse-event records, n (a)	ROR (95% CI)	IC (IC025)
Tamoxifen	SERM	11,373	215	1.13 (0.99 - 1.30)	0.18 (-0.02)
Toremifene	SERM	680	62	5.91 (4.55 - 7.68)	2.45 (1.96)
Fulvestrant	SERD	37,658	712	1.14 (1.05 - 1.23)	0.18 (0.06)
Elacestrant	SERD	2654	25	0.56 (0.38 - 0.83)	-0.83 (-1.37)
Letrozole	AI	65,399	1549	1.46 (1.39 - 1.54)	0.50 (0.42)
Anastrozole	AI	41,536	635	0.91 (0.84 - 0.98)	-0.13 (-0.25)
Exemestane	AI	17,933	385	1.29 (1.17 - 1.43)	0.36 (0.21)

*a*, number of hepatobiliary adverse-event records associated with the target endocrine therapy recorded as the primary suspect (PS) drug; *b*, number of non-hepatobiliary adverse-event records associated with the target endocrine therapy recorded as the PS drug; *c*, number of hepatobiliary adverse-event records associated with other PS drugs within the same restricted reference population; *d*, number of non-hepatobiliary adverse-event records associated with other PS drugs within the same restricted reference population. *a+b* represents the total number of adverse-event records associated with the target endocrine therapy as PS. All four cells were constructed within the same restricted FAERS reference population comprising breast cancer reports from Q1–2004 to Q1–2026 under the PS drug-role definition. No restriction by sex was applied. Hepatobiliary adverse events were defined using the prespecified MedDRA Preferred Term (PT) list provided in Supplementary **Table 2**. ROR, reporting odds ratio; CI, confidence interval; IC, information component; IC025, lower bound of the 95% credible interval.

To evaluate the robustness of the primary findings under alternative analytical definitions, several sensitivity analyses were conducted. In a restricted hepatic-injury analysis based on 16 prespecified PTs, positive signals remained for toremifene [ROR = 4.49, 95% CI: 2.77–7.27; IC = 2.14, IC025 = 1.19], letrozole [ROR = 2.17, 95% CI: 2.01–2.34; IC = 1.02, IC025 = 0.90], and exemestane [ROR = 1.55, 95% CI: 1.32–1.81; IC = 0.61, IC025 = 0.37]. In contrast, the weak positive fulvestrant signal observed with the broad hepatobiliary endpoint was no longer detected [ROR = 0.92, 95% CI: 0.80–1.06; IC = −0.12, IC025 = −0.33]. Tamoxifen, anastrozole, and elacestrant remained below the predefined positive-signal threshold (**Supplementary Table 7**). Drug-role sensitivity analyses showed that the positive signals for toremifene and letrozole were retained across the PS-only, PS+SS, and all-role definitions. For toremifene, the ROR decreased from 5.91 in the PS-only analysis to 5.33 in the PS+SS analysis and 3.26 in the all-role analysis, with corresponding IC025 values of 1.96, 1.90, and 1.30. Letrozole likewise remained positive across all three definitions, with RORs of 1.46, 1.31, and 1.10 and IC025 values of 0.42, 0.27, and 0.06, respectively. Fulvestrant and exemestane remained positive under the PS+SS definition but no longer met the predefined signal criteria when all drug roles were included. Tamoxifen showed a weak positive signal only in the PS+SS analysis, whereas anastrozole and elacestrant remained below the predefined signal threshold under all three definitions (**Supplementary Table 8**).

Concomitant CDK4/6 inhibitor use was particularly frequent among hepatobiliary reports involving fulvestrant [364/516 (70.5%)] and letrozole [748/1,139 (65.7%)] (**Supplementary Table 9**). After exclusion of reports with concomitant CDK4/6 inhibitor use, positive signals were observed for tamoxifen [ROR = 1.18, 95% CI: 1.03–1.35; IC = 0.23, IC025 = 0.03], toremifene [ROR = 5.97, 95% CI: 4.59–7.75; IC = 2.46, IC025 = 2.08], letrozole [ROR = 1.25, 95% CI: 1.15–1.36; IC = 0.30, IC025 = 0.18], and exemestane [ROR = 1.29, 95% CI: 1.16–1.43; IC = 0.35, IC025 = 0.20]. In contrast, the weak positive fulvestrant signal observed in the primary analysis was no longer detected [ROR = 0.96, 95% CI: 0.83–1.10; IC = −0.06, IC025 = −0.27] (**Supplementary Table 10**).

Potential disease- and treatment-related confounding was further evaluated using liver metastasis/hepatic neoplasm indicators and concomitant chemotherapy or HER2-targeted therapy. Metastatic/stage IV breast cancer indicators were most frequently recorded among fulvestrant- [237/516 (45.9%)] and letrozole-associated [437/1,139 (38.4%)] hepatobiliary reports, whereas liver metastasis/hepatic neoplasm indicators were uncommon overall [25/2,630 (1.0%)] (**Supplementary Table 9**). Exclusion of reports containing liver metastasis/hepatic neoplasm indicators did not alter the primary signal classification. After exclusion of reports with concomitant chemotherapy or HER2-targeted therapy, the principal positive signals for toremifene, letrozole, exemestane, and fulvestrant remained positive. Tamoxifen, however, became positive after exclusion of concomitant chemotherapy or HER2-targeted therapy, consistent with the sensitivity observed after exclusion of concomitant CDK4/6 inhibitors. These findings indicate that tamoxifen was sensitive to analytic population definition, whereas the fulvestrant signal was particularly sensitive to concomitant CDK4/6 inhibitor use. In contrast, the positive signals for toremifene, letrozole, and exemestane were comparatively robust across the evaluated disease- and treatment-related exclusions (**Supplementary Table 10**).

### 3.3 Death-related sensitivity analysis

Because death was recorded as an outcome in a subset of hepatobiliary adverse-event reports and may reflect underlying disease severity or malignancy-related factors, a sensitivity analysis was performed to evaluate whether death-recorded cases influenced drug-level disproportionality estimates. Unique case reports with death recorded as an outcome were excluded from the target endocrine-therapy reports, while the original comparator background and predefined signal criteria were retained. After exclusion, disproportionality estimates were recalculated using the same ROR and IC framework as the primary analysis. Positive signals remained for toremifene [ROR = 6.04, 95% CI: 4.65–7.84; IC = 2.47, IC025 = 1.99], letrozole [ROR = 1.41, 95% CI: 1.33–1.49; IC = 0.46, IC025 = 0.37], and exemestane [ROR = 1.28, 95% CI: 1.15–1.42; IC = 0.34, IC025 = 0.18]. In contrast, the weak positive signal observed for fulvestrant in the primary analysis was no longer detected after exclusion of death-recorded cases [ROR = 0.80, 95% CI: 0.73–0.88; IC = −0.31, IC025 = −0.45]. Tamoxifen, anastrozole, and elacestrant remained below the predefined positive-signal threshold (**Supplementary Table 11**). These findings indicate that the main positive signals for toremifene, letrozole, and exemestane were not materially affected by exclusion of death-recorded reports, whereas the fulvestrant signal showed sensitivity to this analytic restriction.

### 3.4 Supplementary pharmacovigilance comparison with VigiAccess

To provide a supplementary pharmacovigilance perspective, aggregated adverse-event term counts were retrieved from VigiAccess on July 7, 2026. Unlike the primary FAERS analysis, which was restricted to breast cancer reports and primary-suspect drugs, the VigiAccess analysis was based on aggregated, indication-unrestricted term-level counts and used the remaining six endocrine therapies as the comparator background. The exact four-cell contingency data and corresponding disproportionality estimates are provided in **Supplementary Table 12**. Toremifene showed the largest disproportionality estimate in VigiAccess [ROR = 5.18, 95% CI: 3.93–6.81; IC = 2.28, IC025 = 1.80]. Tamoxifen also met the predefined positive-signal criteria [ROR = 1.80, 95% CI: 1.69–1.92; IC = 0.66, IC025 = 0.58], and fulvestrant showed a weak positive signal [ROR = 1.09, 95% CI: 1.01–1.18; IC = 0.11, IC025 = 0.01]. In contrast, letrozole [ROR = 0.94, 95% CI: 0.88–0.99; IC = −0.06, IC025 = −0.13], exemestane [ROR = 1.02, 95% CI: 0.93–1.11; IC = 0.02, IC025 = −0.10], anastrozole [ROR = 0.58, 95% CI: 0.53–0.63; IC = −0.66, IC025 = −0.77], and elacestrant [ROR = 0.36, 95% CI: 0.30–0.44; IC = −1.41, IC025 = −1.69] did not meet the predefined positive-signal criteria. The VigiAccess findings were therefore concordant with the FAERS results for the prominent toremifene signal and partially concordant for the weak fulvestrant signal, whereas discordant patterns were observed for tamoxifen, letrozole, and exemestane. Tamoxifen met the predefined positive-signal criteria in VigiAccess but not in the primary FAERS analysis, whereas letrozole and exemestane showed positive signals in FAERS but not in VigiAccess. These discrepancies may reflect differences in indication mix, comparator construction, database composition, reporting practices, drug-role information, and the aggregated nature of VigiAccess data. Accordingly, the VigiAccess findings were interpreted as supplementary descriptive pharmacovigilance evidence rather than as directly comparable or validating estimates (**Supplementary Figure 2**; **Supplementary Table 12**).

### 3.5 PT- and HLT-level hepatobiliary signal patterns

At the PT level, the FAERS-based heatmap demonstrated heterogeneous distributions of hepatobiliary reporting signals across individual endocrine therapies (**Figure 3A**). Broader distributions of detected reporting signals were observed among tamoxifen, toremifene, fulvestrant, and letrozole, whereas elacestrant, anastrozole, and exemestane showed relatively fewer detected PT-level signals. Frequently observed PT-level signals included drug-induced liver injury, hepatic failure, hepatic function abnormal, hepatic steatosis, cholestasis, hepatotoxicity, liver injury, portal vein thrombosis, and steatohepatitis. At the HLT level, heterogeneous signal distributions were also observed across endocrine therapy groups (**Figure 3B**). AI-associated reports showed signals involving hepatocellular injury- and cholestasis-related categories, whereas SERD-associated reports showed signals in selected hepatic failure- and vascular-related categories. SERM-associated reports showed wider confidence intervals across several HLT categories, indicating greater uncertainty due to smaller report numbers. These PT- and HLT-level findings represent exploratory hepatobiliary reporting patterns derived from spontaneous reports and should not be interpreted as differences in clinical incidence, comparative hepatotoxicity, or causal risk.

**Figure 3:**
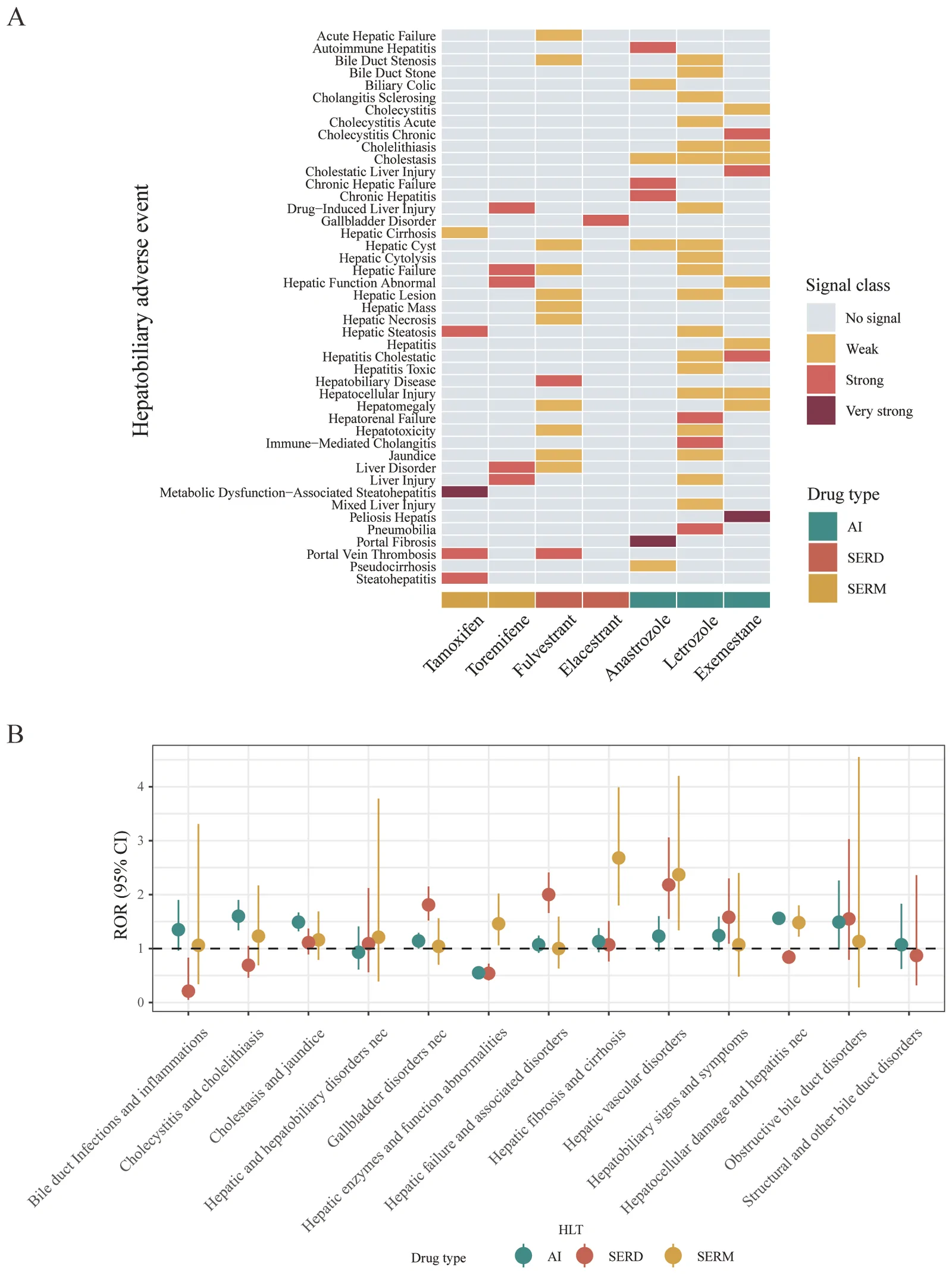
Hepatobiliary disproportionality patterns at the PT and HLT levels. **(A)** PT-level heatmap of hepatobiliary disproportionality signal patterns across seven endocrine therapies. Positive PT-level signals were categorized according to ROR magnitude as weak (ROR <5), strong (ROR 5–30), or very strong (ROR >30); drug–event pairs that did not meet the predefined positive-signal criteria were classified as no signal. **(B)** HLT-level ROR estimates across endocrine therapy classes. Error bars represent 95% confidence intervals. PT, Preferred Term; HLT, high-level term; ROR, reporting odds ratio.

### 3.6 Comparative reporting patterns across endocrine therapy classes

After Benjamini–Hochberg false-discovery-rate correction within each pairwise class comparison, several PT-level differences in hepatobiliary adverse-event reporting patterns remained statistically significant (**Figure 4**). In the AI versus SERD comparison (**Figure 4A**), AI-associated reports showed higher adjusted reporting odds for hepatic cytolysis, drug-induced liver injury, cholestasis, and hepatocellular injury, whereas SERD-associated reports showed higher adjusted reporting odds for hepatic failure, hyperbilirubinaemia, liver disorder, and portal vein thrombosis. In the AI versus SERM comparison (**Figure 4B**), AI-associated reports showed higher adjusted reporting odds for hepatotoxicity, whereas SERM-associated reports showed higher adjusted reporting odds for hepatic steatosis and hepatic cirrhosis. In the SERD versus SERM comparison (**Figure 4C**), SERD-associated reports showed higher adjusted reporting odds for hepatic failure and hepatotoxicity, whereas SERM-associated reports showed higher adjusted reporting odds for hepatic steatosis and hepatic cirrhosis. Detailed PT-level event counts, adjusted odds ratios (ORs) with 95% confidence intervals, raw P values, and BH-adjusted q values for all eligible comparisons are provided in **Supplementary Table 13**. These findings indicate differences in hepatobiliary adverse-event reporting distributions across endocrine therapy classes within FAERS. Given the exploratory nature of these PT-level comparisons and the limitations of spontaneous-reporting systems, the findings should not be interpreted as evidence of differences in clinical incidence, comparative safety, or causal effects.

**Figure 4:**
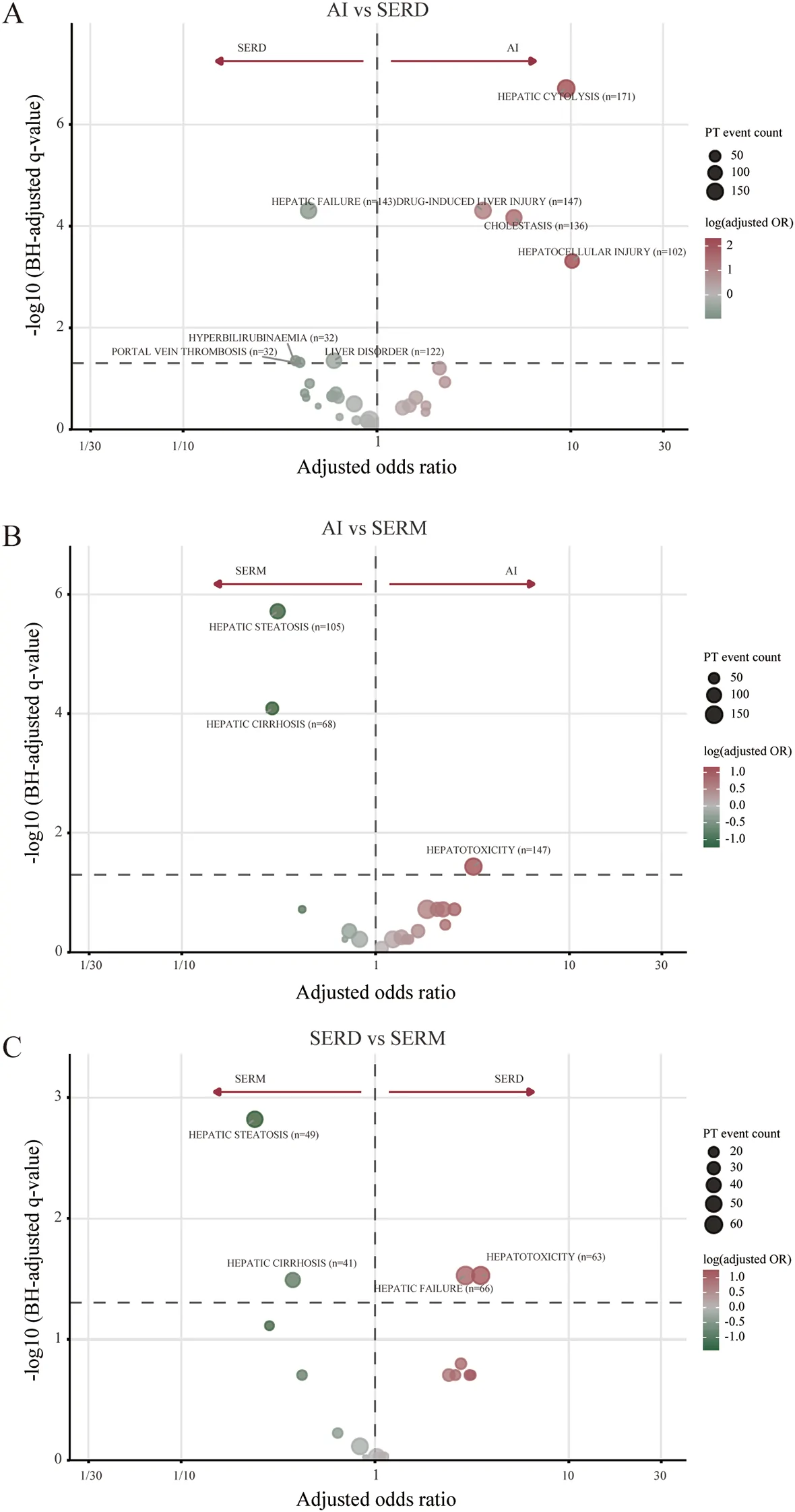
Comparative PT-level reporting patterns across endocrine therapy classes. Volcano plots show adjusted odds ratios for hepatobiliary Preferred Terms (PTs) in pairwise comparisons between endocrine therapy classes: **(A)** AI versus SERD, **(B)** AI versus SERM, and **(C)** SERD versus SERM. Raw P values from PT-specific multivariable logistic regression models were adjusted using the Benjamini–Hochberg false-discovery-rate procedure separately within each pairwise comparison. The y-axis represents −log10(BH-adjusted q value), and the horizontal dashed line indicates q = 0.05. The vertical dashed line indicates an adjusted odds ratio of 1. Only PTs with BH-adjusted q <0.05 are labeled, with the total number of PT events shown in parentheses. Bubble size represents the total number of PT events, and color represents the log-transformed adjusted odds ratio. AI, aromatase inhibitor; SERD, selective estrogen receptor degrader; SERM, selective estrogen receptor modulator.

### 3.7 Factors associated with hepatobiliary adverse-event reporting

In the class-stratified multivariable analyses, age-related hepatobiliary reporting patterns differed across endocrine therapy classes (**Table 3**). Among AI reports, compared with the 45–60-year group, reports involving individuals aged ≤45 years showed higher odds of hepatobiliary adverse-event reporting (adjusted OR = 1.67, 95% CI: 1.36–2.03), whereas those involving individuals aged >60 years showed lower reporting odds (adjusted OR = 0.74, 95% CI: 0.66–0.84). Among SERD reports, compared with the 45–60-year group, the adjusted OR was 1.42 (95% CI: 1.00–1.99) for individuals aged ≤45 years and 0.64 (95% CI: 0.51–0.81) for those aged >60 years. No clear age-related pattern was observed among SERM reports. After multivariable adjustment, neither sex nor concomitant OFS use showed clear associations with hepatobiliary adverse-event reporting in any of the three endocrine therapy classes. These findings describe reporting associations within FAERS and should not be interpreted as patient-level risk factors or causal effects.

**Table 3:** Factors associated with hepatobiliary adverse-event reporting by endocrine therapy class.

Groups/model	Age group (years)			Sex		OFS use status	
	45-60	≤45	>60	Female	Male	No	Yes
	OR (95% CI)			OR (95% CI)		OR (95% CI)	
AIs							
Univariable	Ref	1.70 (1.41-2.05)	0.74 (0.66-0.83)	Ref	0.68 (0.29-1.35)	Ref	1.67 (1.33-2.08)
Multivariable	Ref	1.67 (1.36-2.03)	0.74 (0.66-0.84)	Ref	0.66 (0.28-1.31)	Ref	1.08 (0.84-1.38)
SERDs							
Univariable	Ref	1.43 (1.02-1.98)	0.64 (0.51-0.81)	Ref	0.87 (0.14-2.90)	Ref	1.51 (0.90-2.39)
Multivariable	Ref	1.42 (1.00-1.99)	0.64 (0.51-0.81)	Ref	0.97 (0.16-3.26)	Ref	1.02 (0.59-1.67)
SERMs							
Univariable	Ref	1.05 (0.66-1.63)	1.13 (0.80-1.59)	Ref	1.24 (0.37-3.08)	Ref	0.92 (0.38-1.86)
Multivariable	Ref	1.06 (0.66-1.64)	1.12 (0.79-1.58)	Ref	1.20 (0.36-3.01)	Ref	0.94 (0.39-1.93)

Univariable and multivariable logistic regression analyses were performed separately within each endocrine therapy class. The dependent variable was the presence of at least one prespecified hepatobiliary PT in an individual report (1 = present; 0 = absent); therefore, both hepatobiliary and non-hepatobiliary reports within the same therapy class were included. Age group, sex, and concomitant ovarian function suppression (OFS) use were included simultaneously in the multivariable models. Age 45–60 years, female sex, and no concomitant OFS use were used as the reference categories. Analyses were restricted to complete cases with non-missing age, sex, OFS status, hepatobiliary-event status, and endocrine therapy class information. The numbers of event/non-event reports included in the analyses were 1,449/19,471 for AIs, 367/4,973 for SERDs, and 167/2,678 for SERMs. OR, odds ratio; CI, confidence interval; OFS, ovarian function suppression; AI, aromatase inhibitor; SERD, selective estrogen receptor degrader; SERM, selective estrogen receptor modulator.

### 3.8 Time-to-onset analysis

Among the 2,630 unique hepatobiliary adverse-event reports included in the descriptive cohort, 1,226 (46.6%) contained complete treatment-start and adverse-event onset dates. After excluding 81 reports in which the recorded adverse-event onset preceded treatment initiation, 1,145 reports (43.5% of the cohort) were included in the primary time-to-onset (TTO) analysis (**Supplementary Figure 1**). The availability of evaluable TTO information varied across individual endocrine therapies and therapy classes. At the individual-drug level, TTO completeness ranged from 32.5% for tamoxifen to 68.6% for toremifene, while class-level completeness was 46.4% for aromatase inhibitors (AIs), 34.1% for selective estrogen receptor degraders (SERDs), and 41.5% for selective estrogen receptor modulators (SERMs) (**Supplementary Table 14**). To assess potential selection related to the availability of treatment-start and onset dates, reports included in the TTO analysis were compared with excluded reports. Differences were observed across endocrine therapy class, reporting year, seriousness, reporter country, and reporter type (all P <0.001), with additional differences identified for several hepatobiliary event categories (**Supplementary Table 15**). These findings suggest that evaluable TTO reports may represent a selected subset of the overall reporting population and should therefore be interpreted cautiously.

Among reports with valid TTO information, reported onset distributions differed across endocrine therapy classes (**Figure 5**). The median reported TTO was 64 days (interquartile range [IQR], 28–205 days) for AIs, 82 days (IQR, 26–273 days) for SERDs, and 120 days (IQR, 47–882 days) for SERMs (P = 0.002) (**Table 4**). The cumulative onset curves showed relatively greater accumulation of reported onset times during the earlier period after treatment initiation, although the distributions extended over prolonged intervals, particularly among SERM-associated reports (**Figure 5**). Weibull distribution modeling was subsequently applied to characterize the temporal distribution patterns of reported onset times (**Table 4**). The estimated shape parameters (β) were below 1 for all endocrine therapy classes, including AIs (β = 0.64, 95% CI: 0.61–0.68), SERDs (β = 0.65, 95% CI: 0.58–0.73), and SERMs (β = 0.58, 95% CI: 0.49–0.68). These results were consistent with an early-onset concentration pattern within the available TTO dataset. However, because TTO information was available for only a subset of spontaneous reports and the recorded dates reflect both event occurrence and documentation processes, these Weibull patterns should be interpreted as descriptive distributions of reported onset timing rather than as evidence of biological risk windows or causal temporal relationships.

**Figure 5:**
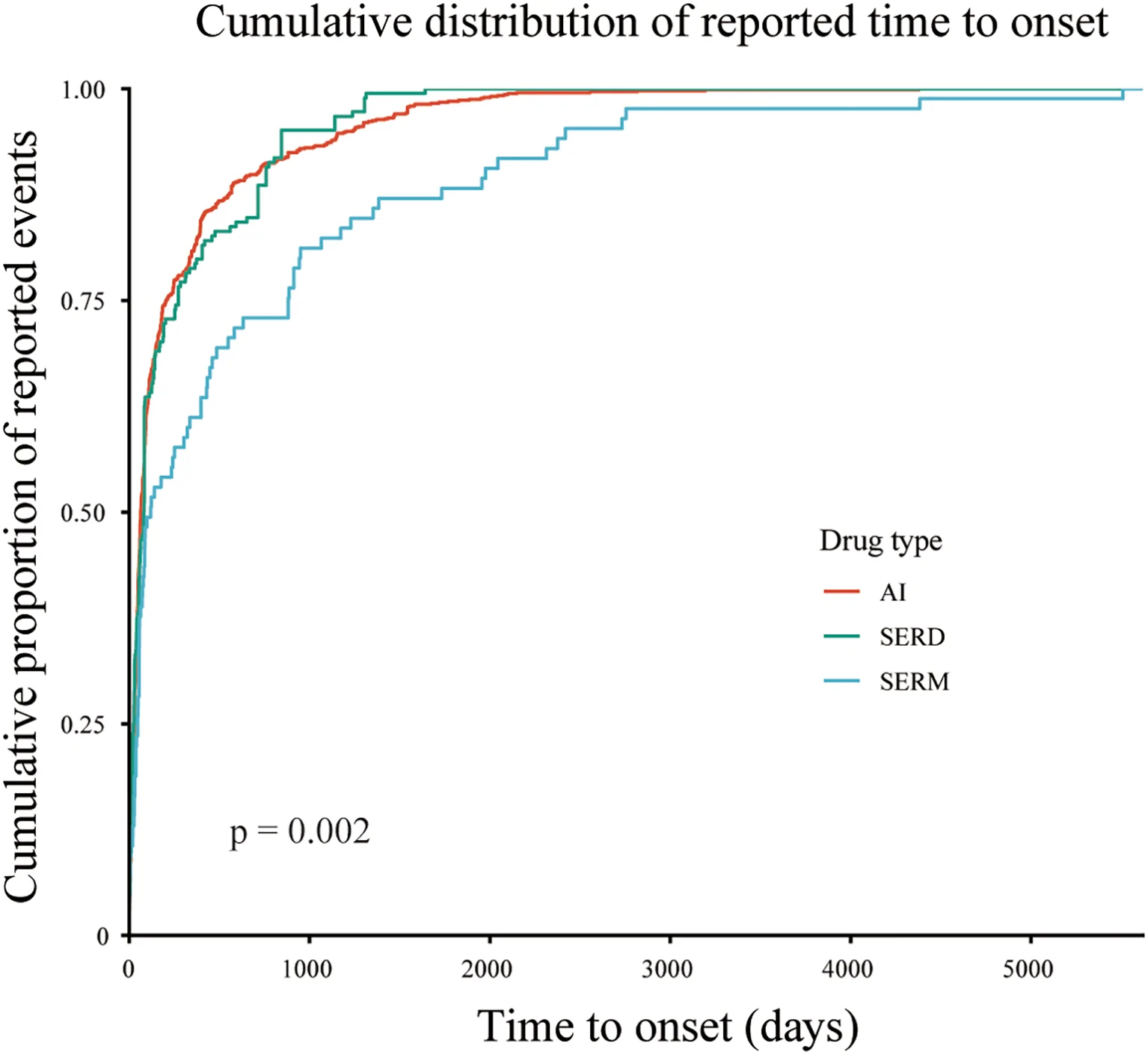
Time-to-onset distribution of hepatobiliary adverse events. Cumulative onset curves show the distributions of reported time to onset across AIs, SERDs, and SERMs among case reports with valid treatment-start and adverse-event onset dates. These curves describe reported onset timing and should not be interpreted as estimates of a biological risk period. AI, aromatase inhibitor; SERD, selective estrogen receptor degrader; SERM, selective estrogen receptor modulator.

**Table 4:** Time-to-onset and Weibull distribution analysis of hepatobiliary adverse events.

Therapy class	Valid TTO reports, n	Median TTO (IQR), days	*P* value	Scale parameter α (95% CI)	Shape parameter β (95% CI)	Weibull pattern
Hepatobiliary disorders						
AI	876	64 (28-205)	0.002	162.05 (145.33-180.71)	0.64 (0.61-0.68)	Early-failure pattern
SERD	184	82 (26-273)		164.16 (129.89-207.48)	0.65 (0.58-0.73)	Early-failure pattern
SERM	85	120 (47-882)		381.99 (259.05-563.28)	0.58 (0.49-0.68)	Early-failure pattern

Time to onset (TTO) was calculated among reports with valid treatment-start and adverse-event onset dates. Median TTO is presented with the interquartile range (IQR). The P value represents the overall comparison of TTO distributions across the three endocrine therapy classes. Weibull parameters were used to characterize the distribution of reported onset times. An upper 95% confidence limit for the shape parameter β <1 was classified as an early-failure pattern according to the prespecified Weibull criterion. Given the incomplete availability of TTO information in spontaneous reports, this classification should be interpreted as a descriptive reporting-time pattern rather than evidence of a biological early-risk period. CI, confidence interval; AI, aromatase inhibitor; SERD, selective estrogen receptor degrader; SERM, selective estrogen receptor modulator.

## 4 Discussion

This pharmacovigilance study characterized hepatobiliary adverse-event reporting patterns for seven endocrine therapies used in breast cancer, using FAERS as the primary data source and VigiAccess as a supplementary exploratory source. The primary FAERS analysis identified heterogeneous drug-level disproportionality patterns. The most consistent positive signals were observed for toremifene, letrozole, and exemestane across multiple sensitivity analyses, whereas tamoxifen and fulvestrant showed more context-dependent patterns under alternative analytic definitions and treatment-related exclusions. PT- and HLT-level analyses further demonstrated heterogeneous reporting patterns across individual drugs and endocrine therapy classes. VigiAccess provided supplementary, non-equivalent pharmacovigilance evidence, with concordant findings for toremifene, partial concordance for fulvestrant, and discordant patterns for tamoxifen, letrozole, and exemestane relative to the primary FAERS analysis. Overall, these findings should be interpreted as exploratory pharmacovigilance reporting patterns rather than evidence of differences in clinical incidence, comparative safety, or causal risk.

Among SERMs, toremifene showed the largest hepatobiliary disproportionality estimate in the primary FAERS analysis, and its signal remained positive across multiple sensitivity analyses. Previous clinical and observational studies have reported fatty liver, nonalcoholic steatohepatitis (NASH), and liver-function abnormalities during toremifene treatment (10, 23–25), while LiverTox has described fatty liver, steatohepatitis, cirrhosis, and rare clinically apparent liver injury with long-term use (26). Tamoxifen showed a more context-dependent reporting pattern, with a borderline drug-level estimate in the primary FAERS analysis, a positive signal in VigiAccess, and a relatively broad PT-level signal spectrum. This pattern is consistent with previous reports of hepatic steatosis, steatohepatitis, nonalcoholic fatty liver disease, and drug-induced liver injury during tamoxifen therapy (27–33), as well as evidence that long-term tamoxifen use may affect fatty liver status (34, 35). However, spontaneous-reporting data lack reliable exposure denominators, and these findings should not be interpreted as evidence of comparative clinical risk.

Among AIs, letrozole and exemestane showed positive hepatobiliary signals in FAERS, whereas anastrozole did not meet the predefined signal criteria. Previous studies have reported liver-function changes and newly developed nonalcoholic fatty liver disease during AI therapy (31, 36, 37), and LiverTox notes that anastrozole, letrozole, and exemestane have been associated with low rates of serum enzyme elevations and rare clinically apparent liver injury (26). The heterogeneity observed among individual AIs may reflect differences in treatment context, patient characteristics, reporting practices, or other unmeasured factors. Among SERDs, fulvestrant showed a weak positive signal that was attenuated under several sensitivity analyses, indicating sensitivity to analytic definition and concomitant treatment context. Elacestrant did not show a positive signal in the primary FAERS analysis, although interpretation is limited by the small number of accumulated hepatobiliary reports; continued post-marketing surveillance is therefore warranted (11). Recent FAERS-based analyses of fulvestrant and elacestrant have likewise demonstrated heterogeneous post-marketing adverse-event reporting patterns, including liver-related events, providing complementary context for the present SERD findings (12).

The supplementary VigiAccess analysis provided a distinct pharmacovigilance perspective but should not be interpreted as external validation of the FAERS findings. Toremifene showed a prominent positive signal in both data sources, while fulvestrant showed only a weak positive pattern in each. In contrast, tamoxifen met the predefined positive-signal criteria in VigiAccess but not in the primary FAERS analysis, whereas letrozole and exemestane showed positive signals in FAERS but not in VigiAccess. These discrepancies likely reflect important methodological differences between the two data sources. The FAERS analysis was restricted to breast cancer reports and primary-suspect drugs and used other PS drugs within the same restricted population as the comparator background, whereas VigiAccess provides aggregated, indication-unrestricted term-level counts and used the remaining six endocrine therapies as the comparator background. Differences in database composition, indication mix, drug-role information, reporting practices, and comparator construction may therefore contribute to the observed discordance. Accordingly, VigiAccess findings are best regarded as supplementary descriptive pharmacovigilance context rather than as directly comparable estimates or confirmatory evidence.

The sensitivity analyses further demonstrated that signal robustness varied across drugs and analytic definitions. Toremifene and letrozole remained positive across restricted-endpoint and broader drug-role analyses, whereas the weaker fulvestrant signal was more sensitive to endpoint definition, drug-role classification, concomitant CDK4/6 inhibitor use, and exclusion of death-recorded cases. Exclusion of reports containing liver metastasis/hepatic neoplasm indicators, concomitant chemotherapy, or HER2-targeted therapy did not materially change the positive signals for toremifene, letrozole, and exemestane. In contrast, tamoxifen showed greater sensitivity to analytic population definition, becoming positive after exclusion of concomitant CDK4/6 inhibitors, chemotherapy, or HER2-targeted therapy, while remaining below the predefined signal threshold after exclusion of liver metastasis/hepatic neoplasm indicators. These findings underscore the attribution challenges inherent in oncology pharmacovigilance, where disease burden, treatment selection, treatment sequence, and multimodal therapy may influence observed reporting patterns. Recent breast cancer data have shown that biomarker profiles may change after neoadjuvant chemotherapy and that such changes may be associated with subsequent clinical outcomes, illustrating how prior treatment and evolving disease biology can alter the clinical context in which later adverse events are reported (38). More broadly, contemporary oncology increasingly involves multimodal treatment strategies combining systemic, targeted, locoregional, and immune-based therapies, further complicating attribution of observed clinical outcomes to a single treatment component (39). Recent studies of intrahepatic cholangiocarcinoma also demonstrate substantial biological heterogeneity within hepatobiliary malignancies, including variation in the immune tumor microenvironment (40). These considerations further support cautious interpretation of liver-related findings in oncology populations. Because explicit disease stage was sparsely recorded and treatment line could not be reliably reconstructed from FAERS, residual confounding by advanced disease status and prior treatment cannot be excluded. Accordingly, these sensitivity analyses support the relative consistency of some reporting signals but cannot fully distinguish drug-related reporting from the surrounding clinical context.

Secondary analyses further explored heterogeneity in hepatobiliary reporting across event categories, patient characteristics, and temporal distributions. These analyses may help characterize the spectrum of reported hepatobiliary events across different therapies; however, class-level comparisons remain exploratory because they may be influenced by treatment allocation, underlying disease characteristics, concomitant therapies, reporting behavior, and other unmeasured factors. Similarly, regression analyses identified age-related variation in reporting patterns among AI- and SERD-associated reports, whereas sex and OFS use showed no consistent associations after adjustment. These findings provide additional characterization of reporting heterogeneity but do not establish differences in clinical susceptibility or biological mechanisms. The TTO analysis also suggested differences in temporal reporting patterns across therapy classes, but incomplete date availability and differences between evaluable and nonevaluable reports limit interpretation. Therefore, TTO findings should be regarded as descriptive characteristics of reported onset timing rather than evidence of biological risk windows.

This study has several strengths and limitations. Strengths include the comprehensive evaluation of seven endocrine therapies using a large pharmacovigilance database, the application of complementary disproportionality approaches, and the incorporation of sensitivity analyses addressing drug-role definitions, concomitant therapies, disease-related factors, and reported outcomes. Several limitations should nevertheless be considered. FAERS is based on spontaneous reports and lacks reliable exposure denominators; therefore, disproportionality estimates cannot be translated into incidence rates or comparative clinical risks. Reporting bias, underreporting, duplicate reporting, stimulated reporting, and differences in clinical practice may also influence observed patterns. In addition, explicit disease stage and treatment line were incompletely recorded and could not be reliably characterized or reconstructed, and residual confounding from treatment selection, disease severity, treatment sequence, and incompletely recorded concomitant medications cannot be fully excluded. Although sensitivity analyses addressed major concomitant anticancer therapies, including CDK4/6 inhibitors, cytotoxic chemotherapy, and HER2-targeted therapy, a comprehensive exclusion analysis covering all potentially hepatotoxic co-medications was not feasible because such medications could not be defined and captured consistently across the full FAERS study period. VigiAccess provides supplementary pharmacovigilance context but does not represent an independent validation dataset because it provides aggregated, indication-unrestricted term-level counts and does not permit equivalent restriction by indication, drug role, concomitant medication, or other individual case-level characteristics. Its comparator background also differed from that used in the primary FAERS analysis; therefore, disproportionality estimates from the two data sources are not directly equivalent. Finally, incomplete TTO information and differences between evaluable and nonevaluable reports may introduce selection bias into temporal analyses. Accordingly, these findings should be interpreted as hypothesis-generating pharmacovigilance evidence requiring confirmation in clinical and epidemiological studies.

## 5 Conclusion

This pharmacovigilance study identified heterogeneous hepatobiliary adverse-event reporting patterns among seven endocrine therapies used for breast cancer. Toremifene, letrozole, and exemestane showed relatively consistent hepatobiliary disproportionality signals across multiple sensitivity analyses, whereas other agents demonstrated more context-dependent patterns. PT- and HLT-level analyses further characterized differences in reported hepatobiliary event distributions across endocrine therapy classes. These findings provide exploratory evidence to prioritize further evaluation of hepatobiliary safety patterns in clinical and epidemiological studies. However, disproportionality signals derived from spontaneous-reporting databases should not be interpreted as estimates of clinical incidence, comparative safety, or causal drug effects.

## Data Availability

The original contributions presented in the study are included in the article/**Supplementary Material**. Further inquiries can be directed to the corresponding authors.

## Supplementary material

Supplementary Figure 1 Flow diagram for derivation of the time-to-onset (TTO) analysis cohort. Among 2,630 hepatobiliary adverse-event reports, 1,226 had complete treatment-start and adverse-event onset dates. After exclusion of 81 reports with negative TTO values, 1,145 reports were included in the primary TTO analysis. Same-day onset (TTO = 0 days; n = 37) was retained in descriptive summaries and treated as 1 day for Weibull fitting. TTO, time to onset; AI, aromatase inhibitor; SERD, selective estrogen receptor degrader; SERM, selective estrogen receptor modulator.

Supplementary Figure 2 Comparison of FAERS- and VigiAccess-based hepatobiliary disproportionality estimates. Forest plots show hepatobiliary disproportionality estimates for seven endocrine therapies. **(A)** Reporting odds ratios (RORs) with 95% confidence intervals. **(B)** Information components (ICs) with IC025–IC975 intervals. “F-” indicates FAERS-based estimates, and “V-” indicates VigiAccess-based estimates. The two analyses used different comparator backgrounds. FAERS estimates were derived from a reference population restricted by breast cancer indication and primary-suspect drug role, with other primary-suspect drugs serving as the comparator background. VigiAccess estimates were derived from aggregated term-level counts and used the remaining six endocrine therapies included in this study as the comparator background. Accordingly, numerical disproportionality estimates from the two data sources should not be interpreted as directly equivalent.
