# Supplementary material for: Hepatobiliary adverse-event reporting patterns for endocrine therapies in breast cancer: a pharmacovigilance study based on FAERS and VigiAccess
Source: Front Endocrinol (Lausanne). 2026 Oct 23;17:1940744. doi: 10.3389/fendo.2026.1940744 (PMC13739581; doi:10.3389/fendo.2026.1940744)
Supplement: Supplementary file 1 [file SupplementaryFile1.docx]

**Supplementary Table 1 Breast cancer-related terms**

| **Breast neoplasms, malignant and unspecified (SMQ) [20000149]** | | | |
| --- | --- | --- | --- |
| **SMQ** | **SMQ_code** | **PT** | **PT_code** |
| Breast malignant tumours (SMQ) | 20000198 | Apocrine breast carcinoma | 10066206 |
| Breast malignant tumours (SMQ) | 20000198 | Breast cancer female | 10006187 |
| Breast malignant tumours (SMQ) | 20000198 | Breast cancer in situ | 10057654 |
| Breast malignant tumours (SMQ) | 20000198 | Breast cancer male | 10006189 |
| Breast malignant tumours (SMQ) | 20000198 | Breast cancer metastatic | 10061020 |
| Breast malignant tumours (SMQ) | 20000198 | Breast cancer recurrent | 10055113 |
| Breast malignant tumours (SMQ) | 20000198 | Breast cancer stage I | 10006198 |
| Breast malignant tumours (SMQ) | 20000198 | Breast cancer stage II | 10006199 |
| Breast malignant tumours (SMQ) | 20000198 | Breast cancer stage III | 10006200 |
| Breast malignant tumours (SMQ) | 20000198 | Breast cancer stage IV | 10006201 |
| Breast malignant tumours (SMQ) | 20000198 | Breast cancer | 10006202 |
| Breast malignant tumours (SMQ) | 20000198 | Breast sarcoma metastatic | 10068582 |
| Breast malignant tumours (SMQ) | 20000198 | Breast sarcoma recurrent | 10068583 |
| Breast malignant tumours (SMQ) | 20000198 | Breast sarcoma | 10068584 |
| Breast malignant tumours (SMQ) | 20000198 | Electron radiation therapy to breast | 10014437 |
| Breast malignant tumours (SMQ) | 20000198 | Extended radical mastectomy | 10015721 |
| Breast malignant tumours (SMQ) | 20000198 | Gamma radiation therapy to breast | 10017677 |
| Breast malignant tumours (SMQ) | 20000198 | HER2 low breast cancer | 10089162 |
| Breast malignant tumours (SMQ) | 20000198 | HER2 negative breast cancer | 10083232 |
| Breast malignant tumours (SMQ) | 20000198 | HER2 positive breast cancer | 10065430 |
| Breast malignant tumours (SMQ) | 20000198 | Hormone receptor negative HER2 positive breast cancer | 10085561 |
| Breast malignant tumours (SMQ) | 20000198 | Hormone receptor positive breast cancer | 10083234 |
| Breast malignant tumours (SMQ) | 20000198 | Hormone receptor positive HER2 negative breast cancer | 10085481 |
| Breast malignant tumours (SMQ) | 20000198 | Hormone refractory breast cancer | 10076935 |
| Breast malignant tumours (SMQ) | 20000198 | Inflammatory carcinoma of breast recurrent | 10021977 |
| Breast malignant tumours (SMQ) | 20000198 | Inflammatory carcinoma of breast stage III | 10021978 |
| Breast malignant tumours (SMQ) | 20000198 | Inflammatory carcinoma of breast stage IV | 10021979 |
| Breast malignant tumours (SMQ) | 20000198 | Inflammatory carcinoma of the breast | 10021980 |
| Breast malignant tumours (SMQ) | 20000198 | Intraductal papillary breast neoplasm | 10073540 |
| Breast malignant tumours (SMQ) | 20000198 | Intraductal proliferative breast lesion | 10073094 |
| Breast malignant tumours (SMQ) | 20000198 | Invasive breast carcinoma | 10075713 |
| Breast malignant tumours (SMQ) | 20000198 | Invasive ductal breast carcinoma | 10073095 |
| Breast malignant tumours (SMQ) | 20000198 | Invasive lobular breast carcinoma | 10073096 |
| Breast malignant tumours (SMQ) | 20000198 | Invasive papillary breast carcinoma | 10073098 |
| Breast malignant tumours (SMQ) | 20000198 | Lobular breast carcinoma in situ | 10073099 |
| Breast malignant tumours (SMQ) | 20000198 | Malignant nipple neoplasm female | 10062051 |
| Breast malignant tumours (SMQ) | 20000198 | Malignant nipple neoplasm male | 10053129 |
| Breast malignant tumours (SMQ) | 20000198 | Malignant nipple neoplasm | 10053128 |
| Breast malignant tumours (SMQ) | 20000198 | Mastectomy | 10026878 |
| Breast malignant tumours (SMQ) | 20000198 | Medullary carcinoma of breast | 10027095 |
| Breast malignant tumours (SMQ) | 20000198 | Metaplastic breast carcinoma | 10073100 |
| Breast malignant tumours (SMQ) | 20000198 | Modified radical mastectomy | 10027799 |
| Breast malignant tumours (SMQ) | 20000198 | Mucinous breast carcinoma | 10073101 |
| Breast malignant tumours (SMQ) | 20000198 | Neuroendocrine breast tumour | 10073103 |
| Breast malignant tumours (SMQ) | 20000198 | Oestrogen receptor assay positive | 10054054 |
| Breast malignant tumours (SMQ) | 20000198 | Paget's disease of nipple | 10033364 |
| Breast malignant tumours (SMQ) | 20000198 | Photon radiation therapy to breast | 10034949 |
| Breast malignant tumours (SMQ) | 20000198 | Postmastectomy lymphoedema syndrome | 10036390 |
| Breast malignant tumours (SMQ) | 20000198 | Primary breast lymphoma | 10081036 |
| Breast malignant tumours (SMQ) | 20000198 | Progesterone receptor assay positive | 10054057 |
| Breast malignant tumours (SMQ) | 20000198 | Radical mastectomy | 10037773 |
| Breast malignant tumours (SMQ) | 20000198 | Radiotherapy to breast | 10062090 |
| Breast malignant tumours (SMQ) | 20000198 | Secretory breast carcinoma | 10088985 |
| Breast malignant tumours (SMQ) | 20000198 | Simple mastectomy | 10040700 |
| Breast malignant tumours (SMQ) | 20000198 | Squamous cell breast carcinoma | 10079307 |
| Breast malignant tumours (SMQ) | 20000198 | Triple negative breast cancer | 10075566 |
| Breast malignant tumours (SMQ) | 20000198 | Triple positive breast cancer | 10083233 |
| Breast malignant tumours (SMQ) | 20000198 | Tubular breast carcinoma | 10073104 |
| Breast malignant tumours (SMQ) | 20000198 | X-ray therapy to breast | 10048199 |
| Breast malignant tumours (SMQ) | 20000198 | Antioestrogen therapy | 10002816 |
| Breast malignant tumours (SMQ) | 20000198 | Breast conserving surgery | 10076783 |
| Breast malignant tumours (SMQ) | 20000198 | Breast reconstruction | 10006305 |
| Breast tumours of unspecified malignancy (SMQ) | 20000199 | Adenomyoepithelioma of breast | 10088008 |
| Breast tumours of unspecified malignancy (SMQ) | 20000199 | Breast neoplasm | 10006279 |
| Breast tumours of unspecified malignancy (SMQ) | 20000199 | Breast tumour excision | 10079326 |
| Breast tumours of unspecified malignancy (SMQ) | 20000199 | Nipple neoplasm | 10056286 |
| Breast tumours of unspecified malignancy (SMQ) | 20000199 | Phyllodes tumour | 10071776 |
| Breast tumours of unspecified malignancy (SMQ) | 20000199 | Biopsy breast abnormal | 10004745 |
| Breast tumours of unspecified malignancy (SMQ) | 20000199 | Breast calcifications | 10048782 |
| Breast tumours of unspecified malignancy (SMQ) | 20000199 | Breast dysplasia | 10006237 |
| Breast tumours of unspecified malignancy (SMQ) | 20000199 | Breast proliferative changes | 10078952 |
| Breast tumours of unspecified malignancy (SMQ) | 20000199 | Computerised tomogram breast abnormal | 10074534 |
| Breast tumours of unspecified malignancy (SMQ) | 20000199 | Wave reflector breast lesion localisation | 10089708 |

**Supplementary Table 3. Preferred Terms included in the restricted 16-PT hepatic-injury sensitivity endpoint.**

| **No.** | **Preferred Term (PT)** | **PT code** | **Clinical category** | **HLT name** | **HLT code** | **Inclusion criterion** |
| --- | --- | --- | --- | --- | --- | --- |
| 1 | CHOLESTASIS | 10008635 | Cholestatic injury | CHOLESTASIS AND JAUNDICE | 10008636 | Direct cholestatic event |
| 2 | CHOLESTATIC LIVER INJURY | 10067969 | Cholestatic injury | HEPATOCELLULAR DAMAGE AND HEPATITIS NEC | 10019833 | Direct cholestatic liver injury |
| 3 | HEPATIC CYTOLYSIS | 10049199 | Hepatocellular injury | HEPATOCELLULAR DAMAGE AND HEPATITIS NEC | 10019833 | Direct hepatocellular injury |
| 4 | DRUG-INDUCED LIVER INJURY | 10072268 | Drug-induced liver injury | HEPATOCELLULAR DAMAGE AND HEPATITIS NEC | 10019833 | Direct drug-induced liver injury term |
| 5 | HEPATIC NECROSIS | 10019692 | Hepatocellular injury | HEPATOCELLULAR DAMAGE AND HEPATITIS NEC | 10019833 | Severe direct hepatic injury |
| 6 | HEPATITIS | 10019717 | Hepatitis / hepatocellular injury | HEPATOCELLULAR DAMAGE AND HEPATITIS NEC | 10019833 | Direct hepatic inflammatory injury |
| 7 | HEPATITIS ACUTE | 10019727 | Hepatitis / hepatocellular injury | HEPATOCELLULAR DAMAGE AND HEPATITIS NEC | 10019833 | Acute direct hepatic inflammatory injury |
| 8 | HEPATITIS CHOLESTATIC | 10019754 | Cholestatic injury | CHOLESTASIS AND JAUNDICE | 10008636 | Cholestatic hepatitis term |
| 9 | HEPATITIS FULMINANT | 10019772 | Hepatitis / hepatocellular injury | HEPATOCELLULAR DAMAGE AND HEPATITIS NEC | 10019833 | Severe acute hepatic injury |
| 10 | HEPATITIS TOXIC | 10019795 | Drug-related hepatic injury | HEPATOCELLULAR DAMAGE AND HEPATITIS NEC | 10019833 | Toxic/drug-related hepatitis term |
| 11 | HEPATOCELLULAR INJURY | 10019837 | Hepatocellular injury | HEPATOCELLULAR DAMAGE AND HEPATITIS NEC | 10019833 | Direct hepatocellular injury |
| 12 | HEPATOTOXICITY | 10019851 | Drug-related hepatic injury | HEPATOCELLULAR DAMAGE AND HEPATITIS NEC | 10019833 | Direct hepatotoxicity term |
| 13 | JAUNDICE CHOLESTATIC | 10023129 | Cholestatic injury | CHOLESTASIS AND JAUNDICE | 10008636 | Hepatic-origin cholestatic jaundice |
| 14 | JAUNDICE HEPATOCELLULAR | 10023136 | Hepatocellular injury | CHOLESTASIS AND JAUNDICE | 10008636 | Hepatocellular-pattern jaundice |
| 15 | LIVER INJURY | 10067125 | Direct liver injury | HEPATOCELLULAR DAMAGE AND HEPATITIS NEC | 10019833 | Direct liver injury term |
| 16 | MIXED LIVER INJURY | 10066758 | Mixed liver injury | HEPATOCELLULAR DAMAGE AND HEPATITIS NEC | 10019833 | Mixed hepatocellular/cholestatic injury |

The restricted hepatic-injury sensitivity endpoint was derived by intersecting the 227 PTs included in the broad MedDRA version 28.1 SOC “Hepatobiliary disorders” endpoint with ACTIVE, NARROW-scope PTs from the Drug related hepatic disorders Standardised MedDRA Query (SMQ). Candidate terms were further restricted to those directly representing drug-induced liver injury, hepatocellular injury/hepatitis, cholestatic liver injury or hepatic-origin jaundice, or mixed liver injury. Nonspecific laboratory abnormalities, structural findings or hepatic masses/lesions, neoplasm- or progression-related manifestations, vascular/coagulation complications, steatotic or metabolic liver disorders, conditions with specific alternative etiologies, and terms denoting suspected rather than directly specified hepatic injury were excluded. The final restricted endpoint comprised 16 PTs.

**Supplementary Table 4. Disproportionality analysis formulas and predefined signal-detection criteria.**

| **Method** | **Calculation formula** | **Criteria** |
| --- | --- | --- |
| ROR | ROR = ad / bc | a ≥ 3 and lower bound of the 95% CI > 1 |
|  | 95% CI = exp[ln(ROR) ± 1.96 × √(1/a + 1/b + 1/c + 1/d)] |  |
| BCPNN | $IC=\log_{2} \left[ \frac{a\left( a+b+c+d \right)}{\left( a+b \right)\left( a+c \right)} \right]$ | IC_025_ > 0 |
|  | 95% credible interval = E(IC) ± 2√V(IC) |  |

ROR, reporting odds ratio; CI, confidence interval; BCPNN, Bayesian Confidence Propagation Neural Network; IC, information component; E(IC), expected value of the information component; V(IC), variance of the information component; IC025, lower bound of the 95% credible interval of the IC. A positive disproportionality signal was defined as *a* ≥ 3, a lower bound of the 95% confidence interval for the ROR > 1, and IC025 > 0. The definitions and exact values of *a*, *b*, *c*, and *d* are provided in Supplementary Table 6.

**Supplementary Table 5. Seriousness, death outcomes, and potential death-related confounding indicators among hepatobiliary adverse-event reports**

| **Drug** | **Hepatobiliary reports, n** | **Serious reports, n (%)** | **Death recorded, n (%)** | **Death reports with malignancy progression/recurrence, n (%)** | **Death reports with hepatic metastasis/neoplasm, n (%)** |
| --- | --- | --- | --- | --- | --- |
| **Tamoxifen** | 154 | 95 (61.7%) | 7 (4.5%) | 2 (28.6%) | 3 (42.9%) |
| **Toremifene** | 51 | 28 (54.9%) | 0 (0.0%) | 0 (NA) | 0 (NA) |
| **Fulvestrant** | 516 | 489 (94.8%) | 160 (31.0%) | 49 (30.6%) | 37 (23.1%) |
| **Elacestrant** | 23 | 22 (95.7%) | 5 (21.7%) | 0 (0.0%) | 0 (0.0%) |
| **Letrozole** | 1,139 | 1,059 (93.0%) | 135 (11.9%) | 79 (58.5%) | 47 (34.8%) |
| **Anastrozole** | 451 | 330 (73.2%) | 42 (9.3%) | 7 (16.7%) | 11 (26.2%) |
| **Exemestane** | 296 | 199 (67.2%) | 32 (10.8%) | 13 (40.6%) | 2 (6.3%) |
| **Overall** | 2,630 | 2,222 (84.5%) | 381 (14.5%) | 150 (39.4%) | 100 (26.2%) |

Seriousness was defined by the presence of at least one coded serious outcome in the FAERS OUTC file. Death recorded indicates that death was reported as an outcome in the case and does not establish that death was caused by the endocrine therapy or the hepatobiliary event. Percentages in the serious and death-recorded columns use all hepatobiliary reports for the corresponding drug as the denominator; percentages in the final two columns use death-reported cases for that drug as the denominator. Malignancy progression/recurrence indicators were identified from recorded indication or adverse-event terms reflecting disease or neoplasm progression/recurrence. Hepatic metastasis/neoplasm indicators included liver metastasis and hepatic malignancy-related terms. NA, not applicable.

**Supplementary Table 6. Four-cell contingency data used in the drug-level disproportionality analyses.**

| **Drug** | **a** | **b** | **c** | **d** | **a+b** | **c+d** | **ROR (95% CI)** | **IC (IC025)** |
| --- | --- | --- | --- | --- | --- | --- | --- | --- |
| **Tamoxifen** | 215 | 11158 | 19124 | 1125122 | 11373 | 1144246 | 1.13 (0.99 - 1.30) | 0.18 (-0.02) |
| **Toremifene** | 62 | 618 | 19277 | 1135662 | 680 | 1154939 | 5.91 (4.55 - 7.68) | 2.45 (1.96) |
| **Fulvestrant** | 712 | 36946 | 18627 | 1099334 | 37658 | 1117961 | 1.14 (1.05 - 1.23) | 0.18 (0.06) |
| **Elacestrant** | 25 | 2629 | 19314 | 1133651 | 2654 | 1152965 | 0.56 (0.38 - 0.83) | -0.83 (-1.37) |
| **Letrozole** | 1549 | 63850 | 17790 | 1072430 | 65399 | 1090220 | 1.46 (1.39 - 1.54) | 0.5 (0.42) |
| **Anastrozole** | 635 | 40901 | 18704 | 1095379 | 41536 | 1114083 | 0.91 (0.84 - 0.98) | -0.13 (-0.25) |
| **Exemestane** | 385 | 17548 | 18954 | 1118732 | 17933 | 1137686 | 1.29 (1.17 - 1.43) | 0.36 (0.21) |

a, number of hepatobiliary adverse-event records associated with the target endocrine therapy recorded as the primary suspect (PS) drug; b, number of non-hepatobiliary adverse-event records associated with the target endocrine therapy recorded as the PS drug; c, number of hepatobiliary adverse-event records associated with other PS drugs within the same restricted reference population; d, number of non-hepatobiliary adverse-event records associated with other PS drugs within the same restricted reference population. a+b represents the total number of adverse-event records associated with the target endocrine therapy as PS, whereas c+d represents the corresponding adverse-event-record comparator background involving other PS drugs. All four cells were constructed within the same restricted FAERS reference population of breast cancer reports from Q1 2004 to Q1 2026 under the PS drug-role definition. No restriction by sex was applied. Hepatobiliary adverse events were defined using the prespecified MedDRA Preferred Term (PT) list provided in Supplementary Table 2. ROR, reporting odds ratio; CI, confidence interval; IC, information component; IC025, lower bound of the 95% credible interval.

**Supplementary Table 7. Sensitivity analysis of drug-level disproportionality using the restricted 16-PT hepatic-injury endpoint.**

| **Drug** | **Class** | **Adverse-event records with the target drug as PS, n (a+b)** | **Restricted 16-PT adverse-event records, n (a)** | **Restricted ROR (95% CI)** | **Restricted IC (IC025)** |
| --- | --- | --- | --- | --- | --- |
| **Tamoxifen** | SERM | 11,373 | 47 | 0.72 (0.54–0.96) | -0.46 (-0.87) |
| **Toremifene** | SERM | 680 | 17 | 4.49 (2.77–7.27) | 2.14 (1.19) |
| **Fulvestrant** | SERD | 37,658 | 197 | 0.92 (0.80–1.06) | -0.12 (-0.33) |
| **Elacestrant** | SERD | 2,654 | 6 | 0.40 (0.18–0.88) | -1.33 (-2.29) |
| **Letrozole** | AI | 65,399 | 753 | 2.17 (2.01–2.34) | 1.02 (0.90) |
| **Anastrozole** | AI | 41,536 | 217 | 0.92 (0.80–1.05) | -0.12 (-0.32) |
| **Exemestane** | AI | 17,933 | 156 | 1.55 (1.32–1.81) | 0.61 (0.37) |

The restricted sensitivity endpoint comprised 16 PTs representing drug-induced liver injury, hepatocellular injury/hepatitis, cholestatic liver injury/hepatic-origin jaundice, and mixed liver injury. All other analytical settings, including the breast cancer indication restriction, primary-suspect drug-role definition, study period, comparator framework, and prespecified signal-detection criteria, were consistent with the primary analysis. A positive signal was defined as a ≥ 3, a lower bound of the 95% confidence interval for the ROR > 1, and IC025 > 0. PS, primary suspect; ROR, reporting odds ratio; CI, confidence interval; IC, information component; IC025, lower bound of the 95% credible interval.

**Supplementary Table 8. Sensitivity analysis of drug-level hepatobiliary disproportionality according to drug-role definition**

| **Drug** | **Class** | **Drug-role definition** | **Adverse-event records with target drug, n (a+b)** | **Hepatobiliary adverse-event records, n (a)** | **ROR (95% CI)** | **IC (IC025)** |
| --- | --- | --- | --- | --- | --- | --- |
| **Tamoxifen** | SERM | PS only | 11,373 | 215 | 1.13 (0.99–1.30) | 0.18 (-0.02) |
|  |  | PS + SS | 39,333 | 739 | 1.13 (1.05–1.22) | 0.17 (0.06) |
|  |  | All roles | 75,156 | 1,198 | 0.95 (0.89–1.01) | -0.07 (-0.16) |
| **Toremifene** | SERM | PS only | 680 | 62 | 5.91 (4.55–7.68) | 2.45 (1.96) |
|  |  | PS + SS | 952 | 79 | 5.33 (4.24–6.72) | 2.31 (1.90) |
|  |  | All roles | 1,700 | 89 | 3.26 (2.63–4.03) | 1.65 (1.30) |
| **Fulvestrant** | SERD | PS only | 37,658 | 712 | 1.14 (1.05–1.23) | 0.18 (0.06) |
|  |  | PS + SS | 76,804 | 1,482 | 1.17 (1.11–1.23) | 0.21 (0.13) |
|  |  | All roles | 129,200 | 2,220 | 1.03 (0.99–1.08) | 0.04 (-0.03) |
| **Elacestrant** | SERD | PS only | 2,654 | 25 | 0.56 (0.38–0.83) | -0.83 (-1.37) |
|  |  | PS + SS | 2,867 | 25 | 0.52 (0.35–0.77) | -0.94 (-1.48) |
|  |  | All roles | 3,000 | 28 | 0.55 (0.38–0.80) | -0.84 (-1.36) |
| **Letrozole** | AI | PS only | 65,399 | 1,549 | 1.46 (1.39–1.54) | 0.50 (0.42) |
|  |  | PS + SS | 134,064 | 2,827 | 1.31 (1.26–1.37) | 0.33 (0.27) |
|  |  | All roles | 216,798 | 3,906 | 1.10 (1.06–1.14) | 0.11 (0.06) |
| **Anastrozole** | AI | PS only | 41,536 | 635 | 0.91 (0.84–0.98) | -0.13 (-0.25) |
|  |  | PS + SS | 62,279 | 1,000 | 0.96 (0.90–1.02) | -0.06 (-0.15) |
|  |  | All roles | 107,025 | 1,530 | 0.84 (0.80–0.88) | -0.23 (-0.30) |
| **Exemestane** | AI | PS only | 17,933 | 385 | 1.29 (1.17–1.43) | 0.36 (0.21) |
|  |  | PS + SS | 43,496 | 830 | 1.15 (1.07–1.23) | 0.19 (0.09) |
|  |  | All roles | 81,189 | 1,373 | 1.01 (0.96–1.07) | 0.02 (-0.07) |

PS-only represents the prespecified primary analysis. Sensitivity analyses were independently rerun using PS+SS and all reported drug-role definitions. For each drug-role definition, target-drug and comparator adverse-event-record counts were recalculated directly from the FAERS data using the same study period, breast cancer indication restriction, hepatobiliary adverse-event definition, comparator framework, and predefined signal-detection criteria as in the primary analysis. ROR and IC estimates were recalculated separately for each drug-role definition. All reported drug roles included primary suspect, secondary suspect, concomitant, and interacting roles. PS, primary suspect; SS, secondary suspect; ROR, reporting odds ratio; CI, confidence interval; IC, information component; IC025, lower bound of the 95% credible interval.

**Supplementary Table 9. Potential disease-burden and concomitant-treatment indicators among hepatobiliary adverse-event reports by endocrine therapy**

| **Drug** | **Hepatobiliary reports, n** | **Metastatic/stage IV marker, n (%)** | **Liver metastasis/ hepatic neoplasm, n (%)** | **Chemotherapy, n (%)** | **HER2-targeted therapy, n (%)** | **Concomitant CDK4/6 inhibitor use, n (%)** |
| --- | --- | --- | --- | --- | --- | --- |
| **Tamoxifen** | 154 | 9 (5.8%) | 0 (0.0%) | 14 (9.1%) | 7 (4.5%) | 3 (1.9%) |
| **Toremifene** | 51 | 2 (3.9%) | 0 (0.0%) | 2 (3.9%) | 0 (0.0%) | 0 (0.0%) |
| **Fulvestrant** | 516 | 237 (45.9%) | 6 (1.2%) | 63 (12.2%) | 18 (3.5%) | 364 (70.5%) |
| **Elacestrant** | 23 | 3 (13.0%) | 0 (0.0%) | 1 (4.3%) | 2 (8.7%) | 3 (13.0%) |
| **Letrozole** | 1,139 | 437 (38.4%) | 11 (1.0%) | 110 (9.7%) | 40 (3.5%) | 748 (65.7%) |
| **Anastrozole** | 451 | 96 (21.3%) | 3 (0.7%) | 39 (8.6%) | 17 (3.8%) | 149 (33.0%) |
| **Exemestane** | 296 | 60 (20.3%) | 5 (1.7%) | 20 (6.8%) | 8 (2.7%) | 31 (10.5%) |

Metastatic/stage IV disease indicators were defined by documented BREAST CANCER METASTATIC or BREAST CANCER STAGE IV indication terms. Liver metastasis/hepatic neoplasm indicators included METASTASES TO LIVER, HEPATIC CANCER METASTATIC, HEPATIC CANCER, and HEPATIC NEOPLASM MALIGNANT. Concomitant chemotherapy, HER2-targeted therapy, and CDK4/6 inhibitor use were identified from recorded concomitant anticancer medications in the DRUG file. Values are report-level counts based on unique PRIMARYID values. The indicators were not mutually exclusive, and a single report could contribute to more than one category. HER2, human epidermal growth factor receptor 2; CDK4/6, cyclin-dependent kinase 4/6.

**Supplementary Table 10. Sensitivity analyses of hepatobiliary disproportionality after exclusion of reports with potential disease- and treatment-related confounding indicators**

| **Exclusion scenario** | **Drug** | **After exclusion, a+b** | **After exclusion, a** | **ROR (95% CI)** | **IC (IC025)** | **Signal** |
| --- | --- | --- | --- | --- | --- | --- |
| **Concomitant CDK4/6 inhibitor exclusion** | **Tamoxifen** | 10,819 | 212 | 1.18 (1.03–1.35) | 0.23 (0.03) | Yes |
|  | **Toremifene** | 674 | 62 | 5.97 (4.59–7.75) | 2.46 (2.08) | Yes |
|  | **Fulvestrant** | 12,675 | 202 | 0.96 (0.83–1.10) | −0.06 (−0.27) | No |
|  | **Elacestrant** | 2,404 | 22 | 0.54 (0.36–0.83) | −0.87 (−1.48) | No |
|  | **Letrozole** | 27,539 | 558 | 1.25 (1.15–1.36) | 0.30 (0.18) | Yes |
|  | **Anastrozole** | 31,855 | 419 | 0.78 (0.71–0.86) | −0.34 (−0.48) | No |
|  | **Exemestane** | 16,345 | 349 | 1.29 (1.16–1.43) | 0.35 (0.20) | Yes |
| **Liver metastasis/hepatic neoplasm exclusion** | **Tamoxifen** | 11,326 | 215 | 1.14 (0.99–1.30) | 0.18 (−0.02) | No |
|  | **Toremifene** | 680 | 62 | 5.91 (4.55–7.68) | 2.45 (1.96) | Yes |
|  | **Fulvestrant** | 37,281 | 704 | 1.14 (1.05–1.23) | 0.17 (0.06) | Yes |
|  | **Elacestrant** | 2,654 | 25 | 0.56 (0.38–0.83) | −0.83 (−1.37) | No |
|  | **Letrozole** | 65,074 | 1,534 | 1.46 (1.38–1.53) | 0.50 (0.42) | Yes |
|  | **Anastrozole** | 41,339 | 632 | 0.91 (0.84–0.98) | −0.13 (−0.25) | No |
|  | **Exemestane** | 17,897 | 380 | 1.28 (1.16–1.42) | 0.34 (0.19) | Yes |
| **Chemotherapy exclusion** | **Tamoxifen** | 9,512 | 197 | 1.23 (1.07–1.42) | 0.29 (0.08) | Yes |
|  | **Toremifene** | 591 | 59 | 6.52 (4.98–8.54) | 2.57 (2.07) | Yes |
|  | **Fulvestrant** | 33,978 | 624 | 1.10 (1.02–1.20) | 0.13 (0.02) | Yes |
|  | **Elacestrant** | 2,607 | 24 | 0.55 (0.36–0.82) | −0.86 (−1.42) | No |
|  | **Letrozole** | 59,023 | 1,393 | 1.45 (1.38–1.54) | 0.50 (0.42) | Yes |
|  | **Anastrozole** | 38,044 | 580 | 0.91 (0.84–0.99) | −0.13 (−0.26) | No |
|  | **Exemestane** | 16,973 | 362 | 1.29 (1.16–1.43) | 0.35 (0.20) | Yes |
| **HER2-targeted therapy exclusion** | **Tamoxifen** | 10,179 | 205 | 1.21 (1.05–1.39) | 0.27 (0.06) | Yes |
|  | **Toremifene** | 654 | 62 | 6.17 (4.75–8.02) | 2.50 (2.02) | Yes |
|  | **Fulvestrant** | 36,445 | 687 | 1.13 (1.05–1.22) | 0.17 (0.06) | Yes |
|  | **Elacestrant** | 2,609 | 23 | 0.52 (0.35–0.79) | −0.92 (−1.49) | No |
|  | **Letrozole** | 61,868 | 1,486 | 1.48 (1.41–1.57) | 0.52 (0.44) | Yes |
|  | **Anastrozole** | 39,486 | 616 | 0.93 (0.86–1.01) | −0.10 (−0.22) | No |
|  | **Exemestane** | 17,347 | 376 | 1.31 (1.18–1.45) | 0.37 (0.22) | Yes |

Sensitivity analyses were performed by excluding target-drug reports containing the specified disease- or treatment-related indicators. After each exclusion, target-drug adverse-event-record counts were recalculated, while the primary-analysis comparator background was retained. ROR and IC estimates were then recomputed using the same predefined signal-detection criteria as in the primary analysis. The exclusion scenarios included concomitant CDK4/6 inhibitor use, liver metastasis/hepatic neoplasm indicators, concomitant chemotherapy, and HER2-targeted therapy. ROR, reporting odds ratio; CI, confidence interval; IC, information component; IC025, lower bound of the 95% credible interval; CDK4/6, cyclin-dependent kinase 4/6; HER2, human epidermal growth factor receptor 2.

**Supplementary Table 11. Sensitivity analysis after exclusion of reports with death recorded as an outcome**

| **Drug** | **Class** | **After exclusion, a+b** | **After exclusion, a** | **ROR (95% CI)** | **IC (IC025)** | **Signal** |
| --- | --- | --- | --- | --- | --- | --- |
| **Tamoxifen** | SERMs | 10,629 | 200 | 1.13 (0.98-1.30) | 0.17 (-0.04) | No |
| **Toremifene** | SERMs | 667 | 62 | 6.04 (4.65-7.84) | 2.47 (1.99) | **Yes** |
| **Fulvestrant** | SERDs | 32,241 | 431 | 0.80 (0.73-0.88) | -0.31 (-0.45) | No |
| **Elacestrant** | SERDs | 2,456 | 20 | 0.48 (0.31-0.75) | -1.04 (-1.64) | No |
| **Letrozole** | AIs | 59,438 | 1,359 | 1.41 (1.33-1.49) | 0.46 (0.37) | **Yes** |
| **Anastrozole** | AIs | 40,109 | 558 | 0.83 (0.76-0.90) | -0.26 (-0.39) | No |
| **Exemestane** | AIs | 16,531 | 350 | 1.28 (1.15-1.42) | 0.34 (0.18) | **Yes** |

Reports with death recorded as an outcome were excluded from target endocrine-therapy reports, while the comparator background used in the primary analysis was retained. The remaining target-drug adverse-event records (a+b) and hepatobiliary adverse-event records (a) were recalculated, followed by ROR and IC estimation using the same primary-analysis framework. A positive signal was defined as a >= 3, a lower limit of the 95% confidence interval for ROR > 1, and IC025 > 0. Death recorded in a FAERS case does not establish that death was caused by the endocrine therapy or the hepatobiliary event. ROR, reporting odds ratio; CI, confidence interval; IC, information component; IC025, lower limit of the 95% credible interval.

**Supplementary Table 12. Four-cell contingency data and disproportionality estimates for the supplementary VigiAccess analysis**

| **Drug** | **a** | **b** | **c** | **d** | **ROR (95% CI)** | **IC (IC025)** |
| --- | --- | --- | --- | --- | --- | --- |
| Tamoxifen | 1,327 | 60,309 | 3,909 | 319,753 | 1.80 (1.69–1.92) | 0.66 (0.58) |
| Toremifene | 55 | 778 | 5,181 | 379,284 | 5.18 (3.93–6.81) | 2.28 (1.80) |
| Fulvestrant | 839 | 56,504 | 4,397 | 323,558 | 1.09 (1.01–1.18) | 0.11 (0.01) |
| Elacestrant | 99 | 19,212 | 5,137 | 360,850 | 0.36 (0.30–0.44) | −1.41 (−1.69) |
| Letrozole | 1,710 | 129,634 | 3,526 | 250,428 | 0.94 (0.88–0.99) | −0.06 (−0.13) |
| Anastrozole | 627 | 72,211 | 4,609 | 307,851 | 0.58 (0.53–0.63) | −0.66 (−0.77) |
| Exemestane | 579 | 41,414 | 4,657 | 338,648 | 1.02 (0.93–1.11) | 0.02 (−0.10) |

VigiAccess data were retrieved on July 7, 2026. For each target endocrine therapy, a represents the aggregated hepatobiliary adverse-event count for the target drug; b represents the aggregated non-hepatobiliary adverse-event count for the target drug; c represents the aggregated hepatobiliary adverse-event count for the remaining six endocrine therapies included in this study; and d represents the aggregated non-hepatobiliary adverse-event count for the remaining six endocrine therapies. Thus, the VigiAccess comparator background consisted of the other endocrine therapies included in this study rather than all medicinal products in VigiAccess. ROR was calculated as ad/bc, with 95% confidence intervals based on the logarithmic approximation. IC denotes the information component, and IC025 denotes the lower bound of the 95% credible interval. Because VigiAccess provides aggregated term-level counts rather than individual case-level reports, these estimates should be interpreted as supplementary exploratory disproportionality measures and are not directly equivalent to the FAERS estimates.

**Supplementary Table 13. PT-level pairwise comparisons between endocrine therapy classes after Benjamini-Hochberg false-discovery-rate correction.**

| **Comparison** | **Preferred Term (PT)** | **Events in first class, n** | **Events in second class, n** | **Total events, n** | **Adjusted OR (95% CI)** | **Raw P value** | **BH-adjusted q value** |
| --- | --- | --- | --- | --- | --- | --- | --- |
| AI vs SERD | ACUTE HEPATIC FAILURE | 33 | 16 | 49 | 0.63 (0.34–1.17) | 0.142 | 0.237 |
| AI vs SERD | BILE DUCT STENOSIS | 8 | 6 | 14 | 0.43 (0.15–1.27) | 0.126 | 0.236 |
| AI vs SERD | CHOLECYSTITIS | 17 | 3 | 20 | 1.78 (0.51–6.19) | 0.364 | 0.455 |
| AI vs SERD | CHOLELITHIASIS | 71 | 12 | 83 | 1.47 (0.79–2.74) | 0.226 | 0.34 |
| AI vs SERD | CHOLESTASIS | 128 | 8 | 136 | **5.08 (2.48–10.43)** | <0.001 | **<0.001** |
| AI vs SERD | DRUG-INDUCED LIVER INJURY | 132 | 15 | 147 | **3.52 (2.06–6.04)** | <0.001 | **<0.001** |
| AI vs SERD | HEPATIC CIRRHOSIS | 46 | 19 | 65 | 0.61 (0.35–1.07) | 0.084 | 0.193 |
| AI vs SERD | HEPATIC CYST | 28 | 14 | 42 | 0.59 (0.30–1.13) | 0.111 | 0.221 |
| AI vs SERD | HEPATIC CYTOLYSIS | 164 | 7 | 171 | **9.47 (4.43–20.24)** | <0.001 | **<0.001** |
| AI vs SERD | HEPATIC FAILURE | 87 | 56 | 143 | **0.44 (0.31–0.63)** | <0.001 | **<0.001** |
| AI vs SERD | HEPATIC FUNCTION ABNORMAL | 101 | 32 | 133 | 0.76 (0.51–1.15) | 0.199 | 0.314 |
| AI vs SERD | HEPATIC LESION | 63 | 23 | 86 | 0.89 (0.55–1.45) | 0.643 | 0.689 |
| AI vs SERD | HEPATIC MASS | 16 | 7 | 23 | 0.78 (0.32–1.94) | 0.597 | 0.663 |
| AI vs SERD | HEPATIC NECROSIS | 16 | 11 | 27 | 0.45 (0.21–0.98) | 0.046 | 0.125 |
| AI vs SERD | HEPATIC PAIN | 9 | 4 | 13 | 0.64 (0.19–2.17) | 0.475 | 0.57 |
| AI vs SERD | HEPATIC STEATOSIS | 69 | 13 | 82 | 1.59 (0.87–2.90) | 0.135 | 0.237 |
| AI vs SERD | HEPATITIS | 69 | 22 | 91 | 0.94 (0.58–1.54) | 0.808 | 0.815 |
| AI vs SERD | HEPATOCELLULAR INJURY | 99 | 3 | 102 | **10.15 (3.21–32.13)** | <0.001 | **<0.001** |
| AI vs SERD | HEPATOMEGALY | 31 | 15 | 46 | 0.59 (0.31–1.12) | 0.106 | 0.221 |
| AI vs SERD | HEPATOTOXICITY | 141 | 57 | 198 | 0.92 (0.67–1.25) | 0.584 | 0.663 |
| AI vs SERD | HYPERBILIRUBINAEMIA | 18 | 14 | 32 | **0.38 (0.18–0.78)** | 0.009 | **0.044** |
| AI vs SERD | HYPERTRANSAMINASAEMIA | 40 | 8 | 48 | 2.24 (1.04–4.82) | 0.039 | 0.117 |
| AI vs SERD | JAUNDICE | 81 | 15 | 96 | 1.36 (0.77–2.38) | 0.289 | 0.377 |
| AI vs SERD | JAUNDICE CHOLESTATIC | 12 | 7 | 19 | 0.42 (0.16–1.11) | 0.079 | 0.193 |
| AI vs SERD | LIVER DISORDER | 82 | 40 | 122 | **0.60 (0.41–0.89)** | 0.01 | **0.044** |
| AI vs SERD | LIVER INJURY | 70 | 12 | 82 | 2.10 (1.13–3.90) | 0.019 | 0.063 |
| AI vs SERD | OCULAR ICTERUS | 6 | 5 | 11 | 0.50 (0.15–1.66) | 0.255 | 0.348 |
| AI vs SERD | PORTAL HYPERTENSION | 25 | 5 | 30 | 1.79 (0.68–4.73) | 0.24 | 0.343 |
| AI vs SERD | PORTAL VEIN THROMBOSIS | 18 | 14 | 32 | **0.40 (0.19–0.82)** | 0.013 | **0.048** |
| AI vs SERD | PSEUDOCIRRHOSIS | 25 | 8 | 33 | 0.91 (0.40–2.04) | 0.815 | 0.815 |
| AI vs SERM | ACUTE HEPATIC FAILURE | 33 | 3 | 36 | 1.49 (0.44–5.03) | 0.524 | 0.607 |
| AI vs SERM | CHOLELITHIASIS | 71 | 8 | 79 | 1.08 (0.51–2.28) | 0.848 | 0.848 |
| AI vs SERM | CHOLESTASIS | 128 | 10 | 138 | 1.23 (0.64–2.38) | 0.539 | 0.607 |
| AI vs SERM | HEPATIC CIRRHOSIS | 46 | 22 | 68 | **0.29 (0.17–0.50)** | <0.001 | **<0.001** |
| AI vs SERM | HEPATIC CYTOLYSIS | 164 | 9 | 173 | 1.84 (0.93–3.66) | 0.08 | 0.19 |
| AI vs SERM | HEPATIC FAILURE | 87 | 10 | 97 | 1.36 (0.69–2.67) | 0.374 | 0.56 |
| AI vs SERM | HEPATIC FUNCTION ABNORMAL | 101 | 20 | 121 | 0.83 (0.50–1.37) | 0.461 | 0.607 |
| AI vs SERM | HEPATIC LESION | 63 | 4 | 67 | 2.55 (0.91–7.17) | 0.075 | 0.19 |
| AI vs SERM | HEPATIC STEATOSIS | 69 | 36 | 105 | **0.31 (0.20–0.48)** | <0.001 | **<0.001** |
| AI vs SERM | HEPATOCELLULAR INJURY | 99 | 5 | 104 | 2.23 (0.90–5.55) | 0.085 | 0.19 |
| AI vs SERM | HEPATOMEGALY | 31 | 4 | 35 | 1.44 (0.49–4.26) | 0.509 | 0.607 |
| AI vs SERM | HEPATOTOXICITY | 141 | 6 | 147 | **3.20 (1.39–7.35)** | 0.006 | **0.037** |
| AI vs SERM | HYPERBILIRUBINAEMIA | 18 | 3 | 21 | 0.69 (0.20–2.46) | 0.573 | 0.607 |
| AI vs SERM | HYPERTRANSAMINASAEMIA | 40 | 3 | 43 | 2.29 (0.70–7.54) | 0.173 | 0.346 |
| AI vs SERM | JAUNDICE | 81 | 7 | 88 | 2.08 (0.94–4.59) | 0.069 | 0.19 |
| AI vs SERM | LIVER DISORDER | 82 | 17 | 99 | 0.73 (0.42–1.27) | 0.269 | 0.441 |
| AI vs SERM | LIVER INJURY | 70 | 6 | 76 | 1.66 (0.70–3.90) | 0.247 | 0.441 |
| AI vs SERM | PORTAL VEIN THROMBOSIS | 18 | 6 | 24 | 0.42 (0.16–1.10) | 0.077 | 0.19 |
| SERD vs SERM | ACUTE HEPATIC FAILURE | 16 | 3 | 19 | 3.07 (0.78–12.11) | 0.109 | 0.197 |
| SERD vs SERM | CHOLELITHIASIS | 12 | 8 | 20 | 1.04 (0.35–3.08) | 0.942 | 0.945 |
| SERD vs SERM | CHOLESTASIS | 8 | 10 | 18 | 0.42 (0.15–1.19) | 0.102 | 0.197 |
| SERD vs SERM | HEPATIC CIRRHOSIS | 19 | 22 | 41 | **0.38 (0.18–0.77)** | 0.008 | **0.032** |
| SERD vs SERM | HEPATIC CYTOLYSIS | 7 | 9 | 16 | 0.29 (0.10–0.84) | 0.023 | 0.077 |
| SERD vs SERM | HEPATIC FAILURE | 56 | 10 | 66 | **2.93 (1.41–6.11)** | 0.004 | **0.03** |
| SERD vs SERM | HEPATIC FUNCTION ABNORMAL | 32 | 20 | 52 | 0.84 (0.44–1.59) | 0.586 | 0.766 |
| SERD vs SERM | HEPATIC LESION | 23 | 4 | 27 | 2.40 (0.78–7.44) | 0.128 | 0.197 |
| SERD vs SERM | HEPATIC STEATOSIS | 13 | 36 | 49 | **0.24 (0.12–0.49)** | <0.001 | **0.002** |
| SERD vs SERM | HEPATOMEGALY | 15 | 4 | 19 | 2.60 (0.76–8.83) | 0.126 | 0.197 |
| SERD vs SERM | HEPATOTOXICITY | 57 | 6 | 63 | **3.51 (1.45–8.48)** | 0.005 | **0.03** |
| SERD vs SERM | HYPERBILIRUBINAEMIA | 14 | 3 | 17 | 3.13 (0.82–11.94) | 0.095 | 0.197 |
| SERD vs SERM | HYPERTRANSAMINASAEMIA | 8 | 3 | 11 | 0.89 (0.22–3.58) | 0.874 | 0.945 |
| SERD vs SERM | JAUNDICE | 15 | 7 | 22 | 2.78 (0.97–7.92) | 0.056 | 0.159 |
| SERD vs SERM | LIVER DISORDER | 40 | 17 | 57 | 1.02 (0.54–1.95) | 0.945 | 0.945 |
| SERD vs SERM | LIVER INJURY | 12 | 6 | 18 | 0.64 (0.22–1.89) | 0.42 | 0.596 |
| SERD vs SERM | PORTAL VEIN THROMBOSIS | 14 | 6 | 20 | 1.11 (0.39–3.18) | 0.849 | 0.945 |

For each comparison, the first class listed in the “Comparison” column was treated as the comparison group and the second class as the reference group. An adjusted OR >1 indicates higher reporting odds in the first class, whereas an adjusted OR <1 indicates higher reporting odds in the second class. Raw P values were adjusted separately within each pairwise comparison using the Benjamini-Hochberg false-discovery-rate procedure. Bold values in the adjusted OR and q-value columns indicate BH-adjusted q <0.05. OR, odds ratio; CI, confidence interval; BH, Benjamini-Hochberg; AI, aromatase inhibitor; SERD, selective estrogen receptor degrader; SERM, selective estrogen receptor modulator.

**Supplementary Table 14. Completeness and drug-specific time-to-onset characteristics of hepatobiliary adverse-event reports**

| **Drug / class** | **Total hepatobiliary reports, n** | **Valid TTO reports, n** | **TTO completeness, %** | **Median TTO, days** | **IQR, days** |
| --- | --- | --- | --- | --- | --- |
| **Individual drugs** | | | | | |
| **Tamoxifen** | 154 | 50 | 32.5 | 517 | 89.8–1,215 |
| **Toremifene** | 51 | 35 | 68.6 | 55 | 28–85 |
| **Letrozole** | 1,139 | 556 | 48.8 | 64.5 | 30.8–181 |
| **Anastrozole** | 451 | 155 | 34.4 | 85 | 24–450 |
| **Exemestane** | 296 | 165 | 55.7 | 56 | 27–184 |
| **Fulvestrant** | 516 | 176 | 34.1 | 81.5 | 25.5–273 |
| **Elacestrant** | 23 | 8 | 34.8 | 72 | 47.8–155.5 |
| **Overall** | **2,630** | **1,145** | **43.5** | **69** | **28–249** |
| **Endocrine therapy classes** | | | | | |
| **AI** | 1,886 | 876 | 46.4 | 64 | 28–205 |
| **SERD** | 539 | 184 | 34.1 | 82 | 26–273 |
| **SERM** | 205 | 85 | 41.5 | 120 | 47–882 |

TTO was calculated as the interval between the recorded treatment start date and adverse-event onset date. Reports with missing or incomplete dates or with a negative interval were excluded. Of 2,630 hepatobiliary reports, 1,226 had complete date pairs; 81 reports with negative TTO values were excluded, leaving 1,145 reports (43.5%) for the primary TTO analysis. Same-day onset (TTO = 0 days; n = 37) was retained in descriptive summaries. Drug-level and class-level completeness differed significantly (both P < 0.001, Pearson chi-square tests). AI, aromatase inhibitor; SERD, selective estrogen receptor degrader; SERM, selective estrogen receptor modulator; TTO, time to onset; IQR, interquartile range.

**Supplementary Table 15. Comparison of reports included versus excluded from the time-to-onset analysis**

| **Characteristic** | **Included in TTO analysis N = 1,145** | **Excluded from TTO analysis N = 1,485** | **P value** |
| --- | --- | --- | --- |
| **Endocrine therapy class** | | | **<0.001** |
| AI | 876 (76.5%) | 1,010 (68.0%) |  |
| SERD | 184 (16.1%) | 355 (23.9%) |  |
| SERM | 85 (7.4%) | 120 (8.1%) |  |
| **Reporting year** | | | **<0.001** |
| 2004–2006 | 75 (6.6%) | 31 (2.1%) |  |
| 2007–2009 | 94 (8.2%) | 47 (3.2%) |  |
| 2010–2012 | 86 (7.5%) | 63 (4.2%) |  |
| 2013–2015 | 44 (3.8%) | 109 (7.3%) |  |
| 2016–2018 | 62 (5.4%) | 165 (11.1%) |  |
| 2019–2021 | 208 (18.2%) | 321 (21.6%) |  |
| 2022–2024 | 374 (32.7%) | 440 (29.6%) |  |
| 2025–2026 | 202 (17.6%) | 309 (20.8%) |  |
| **Seriousness** | | | **<0.001** |
| Serious | 891 (77.8%) | 1,331 (89.6%) |  |
| Non-serious | 254 (22.2%) | 154 (10.4%) |  |
| **Reporter country** | | | **<0.001** |
| United States | 80 (7.0%) | 226 (15.2%) |  |
| United Kingdom | 31 (2.7%) | 88 (5.9%) |  |
| Canada | 4 (0.3%) | 124 (8.4%) |  |
| Germany | 269 (23.5%) | 83 (5.6%) |  |
| Others | 761 (66.5%) | 964 (64.9%) |  |
| **Reporter type** | | | **<0.001** |
| Consumer | 123 (10.7%) | 284 (19.1%) |  |
| Health professional | 145 (12.7%) | 186 (12.5%) |  |
| Pharmacist | 124 (10.8%) | 97 (6.5%) |  |
| Physician | 615 (53.7%) | 710 (47.8%) |  |
| Missing/other | 138 (12.1%) | 208 (14.0%) |  |
| **Hepatobiliary event category** | | |  |
| Hepatocellular damage/hepatitis | 602 (52.6%) | 698 (47.0%) | 0.005 |
| Hepatic failure | 172 (15.0%) | 153 (10.3%) | <0.001 |
| Cholestasis/jaundice | 203 (17.7%) | 214 (14.4%) | 0.024 |
| Fibrosis/cirrhosis | 43 (3.8%) | 121 (8.1%) | <0.001 |
| Hepatic vascular disorders | 27 (2.4%) | 69 (4.6%) | 0.003 |
| Biliary disorders | 132 (11.5%) | 149 (10.0%) | 0.243 |
| Hepatic enzyme/function abnormalities | 105 (9.2%) | 138 (9.3%) | 0.968 |
| Other hepatobiliary | 150 (13.1%) | 342 (23.0%) | <0.001 |

Data are presented as n (%). Reports were included in the TTO analysis when both treatment start and adverse-event onset dates were complete and the calculated interval was non-negative; all remaining hepatobiliary reports were classified as excluded. Overall P values for endocrine therapy class, reporting year, seriousness, reporter country, and reporter type were calculated using Pearson chi-square tests. Hepatobiliary event categories were defined from MedDRA HLT groupings and were not mutually exclusive; therefore, each event category was compared separately as present versus absent. Serious reports were defined according to the outcome coding used in the primary descriptive analysis. TTO, time to onset; AI, aromatase inhibitor; SERD, selective estrogen receptor degrader; SERM, selective estrogen receptor modulator; HLT, high-level term.
